# Lost lovers linked at long last: elusive female *Nanophyllium* mystery solved after a century of being placed in a different genus (Phasmatodea, Phylliidae)

**DOI:** 10.3897/zookeys.969.56214

**Published:** 2020-09-17

**Authors:** Royce T. Cumming, Stéphane Le Tirant, Sierra N. Teemsma, Frank H. Hennemann, Luc Willemse, Thies H. Büscher

**Affiliations:** 1 Associate Researcher, Montreal Insectarium, 4581 rue Sherbrooke est, Montréal, Québec, H1X 2B2, Canada Montreal Insectarium Montréal Canada; 2 Ph.D. Student, Richard Gilder Graduate School, American Museum of Natural History, New York, NY 10024, USA American Museum of Natural History New York United States of America; 3 Ph.D. program in Biology, Graduate Center, City University of New York, NY, USA City University of New York New York United States of America; 4 Collection manager, Montreal Insectarium, 4581 rue Sherbrooke, Montréal, Québec, H1X 2B2, Canada Montreal Insectarium Montréal Canada; 5 Tannenwaldallee 53, 61348 Bad Homburg, Germany Unaffiliated Bad Homburg Germany; 6 Naturalis Biodiversity Center, PO Box 9517, NL-2300 RA Leiden, The Netherlands Naturalis Biodiversity Center Leiden Netherlands; 7 Department of Functional Morphology and Biomechanics, Zoological Institute, Kiel University, Am Botanischen Garten 9, 24118 Kiel, Germany Kiel University Kiel Germany

**Keywords:** Nano-leaf insect, Phasmida, Phylliinae, *Phyllium*, sexual dimorphism, taxonomy, walking leaf, West Papua

## Abstract

After successful laboratory rearing of both males and females from a single clutch of eggs, the genus *Nanophyllium* Redtenbacher, 1906 (described only from males) and the *frondosum* species group within Phyllium (Pulchriphyllium) Griffini, 1898 (described only from females) are found to be the opposite sexes of the same genus. This rearing observation finally elucidates the relationship of these two small body sized leaf insect groups which, for more than a century, have never been linked before. This paper synonymizes the *frondosum* species group with *Nanophyllium* Redtenbacher, 1906 in order to create a singular and clearly defined taxonomic group. Five species are transferred from the Phyllium (Pulchriphyllium) frondosum species group and create the following new combinations: *Nanophylliumasekiense* (Größer, 2002), **comb. nov.**; *Nanophylliumchitoniscoides* (Größer, 1992), **comb. nov.**; *Nanophylliumfrondosum* (Redtenbacher, 1906), **comb. nov.**; *Nanophylliumkeyicum* (Karny, 1914), **comb. nov.**; *Nanophylliumsuzukii* (Größer, 2008), **comb. nov.** The only taxon from this species group not transferred from the *frondosum* species group to *Nanophyllium* is Phyllium (Pulchriphyllium) groesseri Zompro, 1998. Based on protibial exterior lobes, this species belongs in the *schultzei* species group as described in Hennemann et al. 2009 and is therefore excluded from further discussion here. The rearing of *Nanophyllium* also yielded the male *Nanophylliumasekiense* (Größer, 2002), **comb. nov.** thus, enabling comparison of this male to the other previously known *Nanophyllium* species. Two new species of nano-leaf insects are described within, *Nanophylliummiyashitai***sp. nov.**, from Morobe Province, Papua New Guinea, and *Nanophylliumdaphne***sp. nov.**, from Biak Island, Papua Province, Indonesia. With such distinct sexual dimorphism in *Nanophyllium* between sexes, which have only now been matched up via captive rearing, illustrated within are numerous specimens which might represent the unknown opposite sexes of the many currently known species of *Nanophyllium*. Due to pronounced sexual dimorphism in *Nanophyllium*, only future captive rearing or molecular analysis will match up the many unknown sexes. To conclude, with the description of two new *Nanophyllium* species, dichotomous keys to species for known males and females are presented.

## Introduction

**Figure 1.**
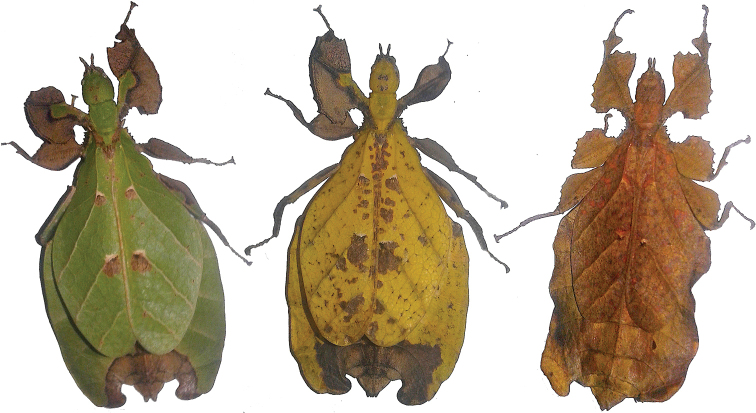
Live *Nanophylliumasekiense* (Größer, 2002), comb. nov. females, three primary color forms photographed in Morobe Province, Papua New Guinea.

The leaf insects (Phylliidae) represent a lineage within the stick insects (Phasmatodea), which are popular as pets and within insect collections due to their impressive leaf-like camouflage. The Phylliidae have an impressive array of morphological adaptations to mimic different types of leaves. Present in many species is the ability for various color forms which allow them to look like living, dying, or dead leaves (Fig. 1). These masters of leaf-like camouflage can be found throughout Southeast Asia, with some of the biodiversity hotspots being Indonesia, Malaysia, and Papua New Guinea (Brock et al. 2020).

Included within the Phylliidae are the *Nanophyllium* Redtenbacher, 1906 a group of small bodied species which for more than 100 years have only been known from rarely collected male specimens. Throughout a majority of those years only the single type species *Nanophylliumpygmaeum* Redtenbacher, 1906 was recognized. It was not until the last couple of decades that a majority of new *Nanophyllium* species began to be described (Brock et al. 2020). With numerous species described throughout the island of New Guinea solely from male specimens, the question begs to be asked, “where are the female *Nanophyllium*?”. Could the lack of females be that they are incredibly elusive, or already well-known and simply not recognized as female *Nanophyllium*?

Brock and Hasenpusch (2003: 203, fig. 6) were the first to suggest and illustrate the unknown *Nanophyllium* female, an individual from Nabire, Irian Jaya, collected along with a male which would later become the holotype of *Nanophylliumhasenpuschi* Brock & Größer, 2008. Unfortunately, however, the female specimen is missing both front legs (an important morphological identification feature frequently used for differentiation) but all other features place it morphologically close to Phyllium (Pulchriphyllium) frondosum Redtenbacher, 1909. Additionally, Brock and Hasenpusch (2003) discuss the subadult female found by G. B. Monteith in 1971 in the Iron Range, Queensland as additional evidence that a “*frondosum*-like species” is probably the unknown female for the *Nanophyllium*. As it turns out, the *frondosum* species group, as defined by Hennemann et al. (2009), are also somewhat of a quandary since they are only known from female specimens, except for Phyllium (Pulchriphyllium) groesseri Zompro, 1998. In light of the speculation by Brock and Hasenpusch (2003) the authors scoured major collections around the world for additional *Nanophyllium* male specimens and females of the *frondosum* species group looking for shared morphological features which might support the thoughts of Brock and Hasenpusch (2003).

As it so happens, the breakthrough was not due to the exhaustive review of museum specimens, but instead came as a surprise at the Montreal Insectarium, Quebec, Canada. In April 2018 the Insectarium received eggs of Phyllium (Pulchriphyllium) asekiense Größer, 2002 from Morobe Province, Papua New Guinea with the hopes of raising this beautifully variable species in captivity (Fig. 1). The surprise came in early 2019 when the three sole survivors of the very slow developing nymphs reached adulthood; as one female *Phylliumasekiense* and two male ‘*Nanophyllium*’. This breakthrough at long last was the evidence we had been searching for to remove the speculation of these two known, but never associated, taxa.

This pairing of the *frondosum* species group with the *Nanophyllium* morphologically was not surprising given numerous morphological similarities between the taxonomic groups (discussed below). From our extensive review of museum specimens, we found males ranging in length from ~ 27 to 40 mm and females ranging from ~ 46 to 71 mm, a difference which is not outside of the norm with the male to female ratio seen throughout the family.

## Materials and methods

The holotype specimen of *Nanophylliummiyashitai* sp. nov. was loaned to the Montreal Insectarium (Stéphane Le Tirant, collection manager) from the extensive collection of Tetsuo Miyashita, Japan. See the abbreviations section below for a full list of collections (both institutional and large private) in which relevant material was recovered.

Photos of specimens that were loaned to/held within the Montreal Insectarium were taken by René Limoges of using a Nikon D810 DSLR camera with Nikon Micro-Nikkor 200 mm f/4 lens on Manfrotto 454 micrometric positioning sliding plate. Lighting was provided by two Nikon SB-25 flash units with a Cameron Digital diffusion photo box. Adobe Photoshop Elements 13 was used as post processing software. The *Nanophylliummiyashitai* sp. nov. holotype specimen is deposited in the Montreal Insectarium (Quebec, Canada) type collection.

Photographs of the female *Nanophylliumchitoniscoides* (Größer, 1992) comb. nov. (Fig. 16A) were taken by Frank Hennemann within his personal collection using a Nikon D7000 camera equipped with a Nikon DX AF-S Micro 40 mm lens and a wireless Nikon SU-800 dual speed light system. Background lightning was provided by a 18W 6000K LED panel light plate.

The photograph of the female holotype *Nanophylliumsuzukii* (Größer, 2008) comb. nov. (Fig. 16B) was taken by Mandy Schröter under direction of Stephan Blanke at the Senckenberg German Entomological Institute Müncheberg using a Nikon D7200 digital camera and a Nikon Micro Nikkor 105 mm f/2.8 G ED objective. Lightning was from the Yongnuo Digital Speedlight YN 560 IV reflected by the inner surface of a Styrofoam box set up around the specimen. A grey card was used for white balance. Composite images with an extended depth of field were created using the software StackShot Macro Rail Package (Cognisys Inc., U.S.A.) and Zerene Stacker (release November 7, 2017; Zerene Systems LLC, U.S.A.).

Photographs of the types of *Nanophylliumdaphne* sp. nov. (Fig. 21C) and *Nanophylliumkeyicum* (=*Phylliuminsulanicum*) (Fig. 16D) were taken by Yvonne van Dam at Naturalis using a Nikon D600 with a 60 mm macro lens.

All other photographs were taken by unknown photography equipment/by simple camera phone images, and where photo credit/equipment is known it is stated within the figure caption. Egg orientation terminology follows Clark (1978). Species group organizations follow the classification presented in Hennemann et al. (2009) and Cumming (2017). Wing venation terminology follows Burt (1932) and Ragge (1955). Measurements of the holotype specimens were made to the nearest 0.1 mm using digital calipers.

The following institutional abbreviations are used:

**AMNH** American Museum of Natural History, New York, New York, U.S.A.

**ANIC** Australian National Insect Collection, Canberra, Australia

**ANSP** Academy of Natural Sciences, Philadelphia, Pennsylvania, U.S.A.

**CAS** California Academy of Sciences, San Francisco, California, U.S.A.

**CFIA** California Academy of Sciences, San Francisco, California, U.S.A.

**IMQC** Insectarium de Montréal, Montréal, Québec, Canada

**MNHU** Museum für Naturkunde der Humboldt-Universität, Berlin, Germany

**MSNG** Museo Civico di Storia Naturale, Genova, Italy

**MZSF** Strasbourg Zoological Museum, Strasbourg, France

**NHMB** Naturhistorisches Museum Basel, Basel, Switzerland

**NHMUK** Natural History Museum United Kingdom, London, United Kingdom

**RBINS** Royal Belgian Institute of Natural Sciences, Brussels, Belgium

**RMNH** Naturalis Biodiversity Center, Leiden, Netherlands

**SDEI** Senckenberg Deutsches Entomologisches Institut, Müncheberg, Germany

**SDNHM** San Diego Natural History Museum, San Diego, California, U.S.A.

**SMTD** Staatliches Museum für Tierkunde, Dresden, Germany

**UMMZ** University of Michigan, Museum of Zoology, Ann Arbor, Michigan, U.S.A.

**Coll FH** Private collection of Frank H. Hennemann, Bad Homburg, Germany

**Coll RC** Private collection of Royce T. Cumming, California, U.S.A.

**Coll SLT** Private collection of Stéphane Le Tirant, Montreal, Canada

## Results

### Captive rearing of Nanophyllium in the Montreal Insectarium

**Figure 2.**
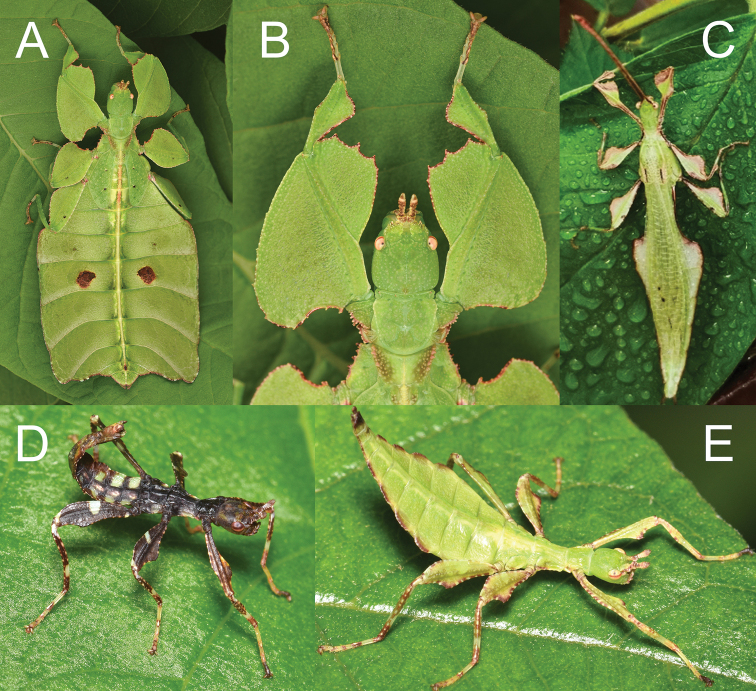
Live *Nanophylliumasekiense* (Größer, 2002), comb. nov. raised in the Montreal Insectarium **A** subadult female, full body dorsal **B** subadult female, head and front legs, dorsal **C** adult male, full body dorsal **D** freshly hatched nymph **E** nymph, later instar.

In January 2018, Stéphane Le Tirant of the Montreal Insectarium applied for a live insect import permit from the CFIA to allow the importation of live eggs to Canada from local insect suppliers in Papua New Guinea. Fortunately, one of those suppliers was able to export a small series of eggs freshly laid by a wild caught female *Phylliumasekiense*, collected in Morobe Province, Papua New Guinea. In April 2018, the Montreal Insectarium received 13 eggs, all black in color, and with the same morphology across the series. Eggs were incubated at room temperature on a plastic screen mesh within a plastic box with coco fiber at the base which was regularly sprayed with water to maintain humidity. Of these thirteen received eggs, during a period of seven to eleven months only five nymphs hatched (Fig. 2D). In an attempt to find at least one species of food which the fresh nymphs would accept, nymphs were offered *Psidiumguajava* (Guava), *Rubus* sp. (Bramble), and *Gaultheriashallon* (Salal) to eat. These three plants are commonly accepted host plants regularly used within the Montreal Insectarium.

Of these five nymphs, two nymphs refuse to eat any of the host plants offered entirely and died within a few days. The other three nymphs thankfully accepted bramble and during the first few instars all three individuals looked very similar in morphology (Fig. 2E). All three nymphs were quite active both day and night and fed continuously. It was not until the last two molts that the sexual dimorphism became noticeable, and after a period of 90 to 130 days after first hatching all three reached adulthood as two males and one female. Unfortunately, the males became adult (Fig. 2C) whilst the female was still only a subadult (Fig. 2A). The males were active by day and night (readily flying) and lived only for about four months, dying before the female was adult.

**Figure 3.**
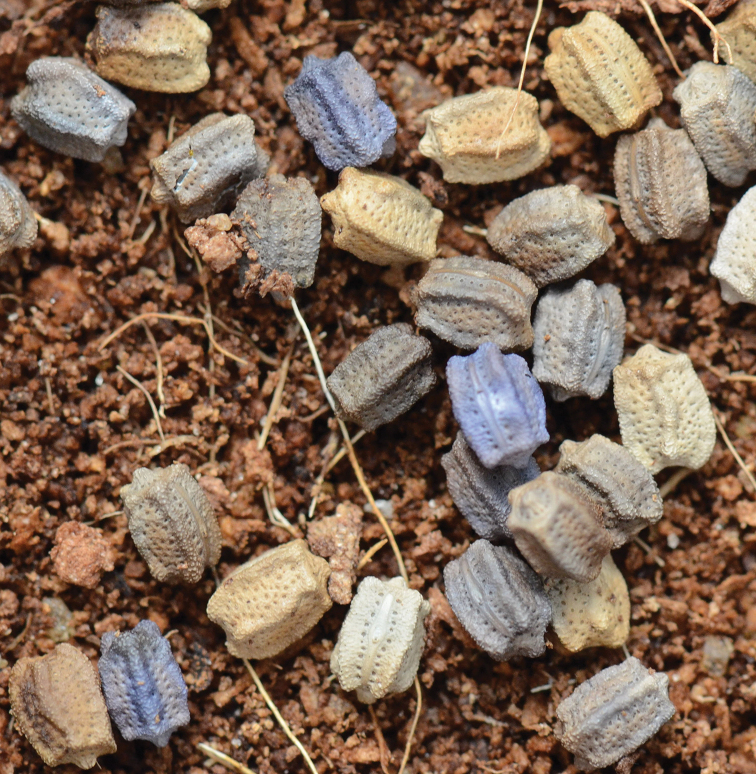
*Nanophylliumasekiense* (Größer, 2002), comb. nov. eggs laid by the single adult female which made it to adulthood in the Montreal Insectarium; note the variability of color within the eggs laid by this singular female.

The female lived as an adult for about nine months before dying, producing a total of 245 eggs of many different colors (Fig. 3). During the peak of her egg producing life she produced three to four eggs per day, and as many as 112 eggs in a single month. In adulthood, the female was no longer active during the day and only fed at night. In the months since the 245 eggs were laid, they have been incubating and during this period very few nymphs have successfully hatched, and all refused to feed on the three host plant species that were offered and accepted by the original culture. To try and ensure that the species was successfully brought into culture, the Montreal Insectarium shared many eggs with other experienced Phylliidae breeders but they also were unsuccessful with obtaining a subsequent generation.

Despite the failed attempt to bring this species into culture, the successful rearing of both sexes from a batch of eggs from a single female has allowed the much needed definitive evidence to back up the morphological observations that the females of the *frondosum* species group and the males of *Nanophyllium* are simply one and the same. Therefore, the *frondosum* species group of Phyllium (Pulchriphyllium) is here transferred to the genus *Nanophyllium* (except for *Ph.groesseri*, which instead belongs to the Phyllium (Pulchriphyllium) schultzei species group based in the protibial lobes and is therefore left within the Phyllium (Pulchriphyllium) subgenus). The genus *Nanophyllium* is here revised with most species illustrated and a general description for the male, female, and egg morphology after more than 100 years is now summarized together.

### Taxonomic accounts

#### *Nanophyllium* Redtenbacher, 1906: 180.

**Type species.***Nanophylliumpygmaeum* Redtenbacher, 1906, by monotypy.

**Figure 4.**
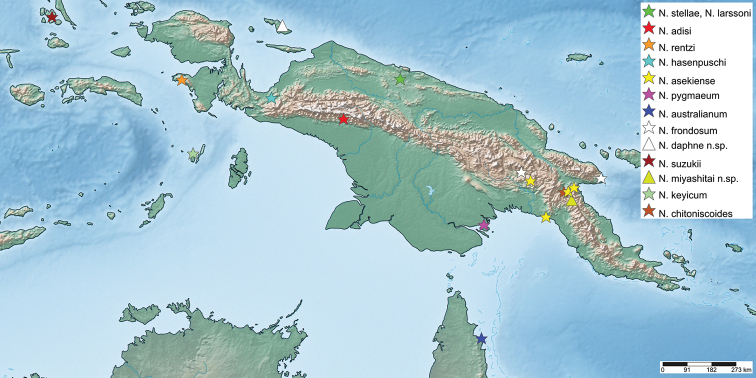
Distribution map showing localities for the known and herein described species.

**Distribution**. East of Weber’s biogeographic line of faunal balance (Gressitt, 1982), primarily on the island of New Guinea and surrounding islands, as well as on the northern tip of Australia (Fig. 4).

#### Species group checklist and sex which is known

*pygmaeum* species group

*Nanophylliumadisi* Zompro & Größer, 2003 (Male)

*Nanophylliumasekiense* (Größer, 2002) comb. nov. (Female, Egg, Male)

*Nanophylliumaustralianum* Cumming, Le Tirant, & Teemsma, 2018 (Male)

*Nanophylliumchitoniscoides* (Größer, 1992) comb. nov. (Female, Egg)

*Nanophylliumfrondosum* (Redtenbacher, 1906) comb. nov. (Female)

*Nanophylliumhasenpuschi* Brock & Größer, 2008 (Male)

*Nanophylliumkeyicum* (Karny, 1914) comb. nov. (Female, Egg)

*Nanophylliumpygmaeum* Redtenbacher, 1906 (Male)

*Nanophylliumrentzi* Brock & Größer, 2008 (Male)

*Nanophylliumsuzukii* (Größer, 2008) comb. nov. (Female, Egg)

*Nanophylliumdaphne* Cumming, Willemse, Le Tirant, Teemsma, Hennemann and Büscher, sp. nov. (Female)

*stellae* species group

*Nanophylliumstellae* Cumming, 2016 (Male)

*Nanophylliumlarssoni* Cumming, 2017 (Male)

*Nanophylliummiyashitai* Cumming, Le Tirant, Teemsma, Hennemann, Willemse and Büscher, sp. nov. (Male)

#### Female *Nanophyllium* general morphology

**Figure 5.**
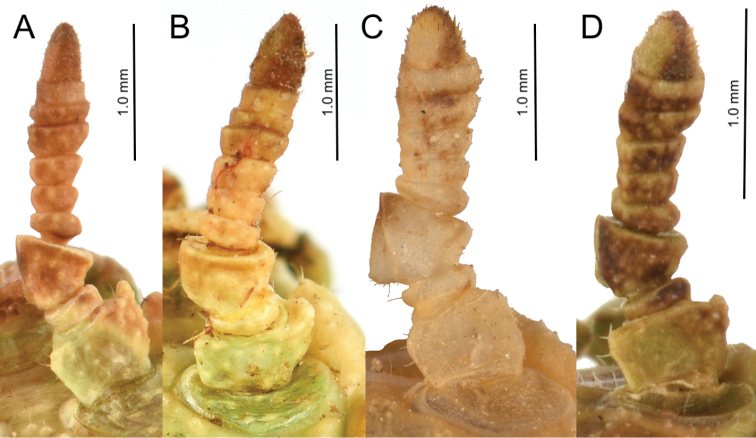
Female *Nanophyllium* antennae, dorsal view **A***Nanophylliumasekiense*Coll SLT**B***Nanophyllium* species, Indonesia, West Papua, Mapia, Coll SLT**C***Nanophylliumdaphne* sp. nov., RMNH**D***Nanophyllium* species NHMUK 012497230.

**Figure 6.**
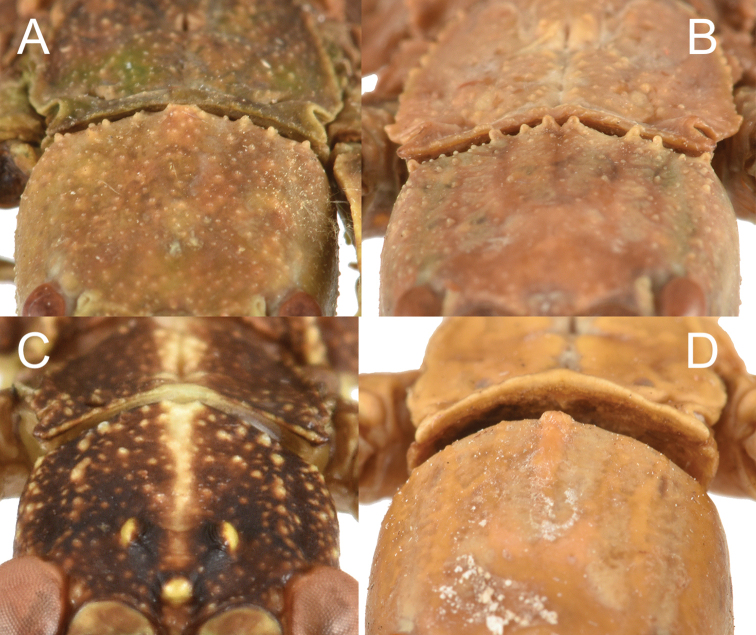
Head capsule posteriomedial tubercles compared between select genera/species **A***Nanophyllium* species female NHMUK 012497230 **B***Nanophylliumfrondosum* female, Coll SLT**C***Nanophylliummiyashitai* sp. nov. holotype male **D***Phylliumletiranti* Cumming & Teemsma, 2018 female, Coll SLT (note the singular tubercle in this *Phyllium* species versus the double tubercles of *Nanophyllium*).

**Figure 7.**
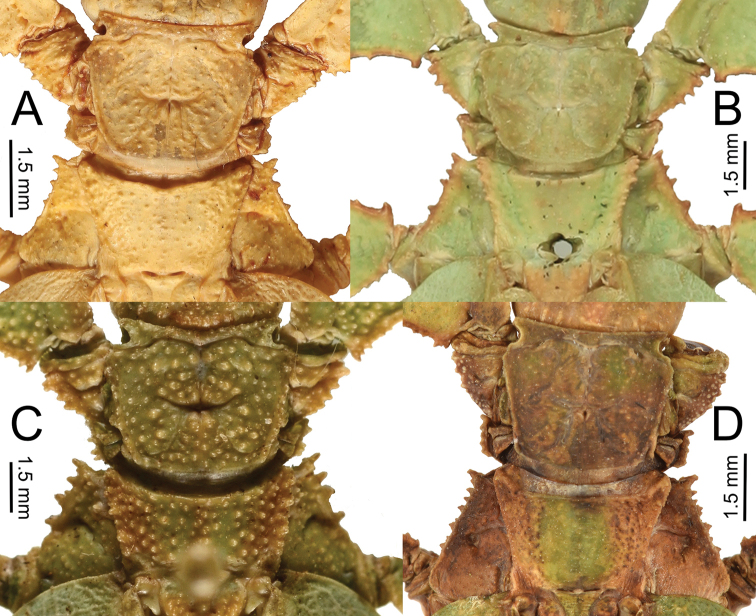
Thorax of various *Nanophyllium* females, dorsal **A***Nanophyllium* species female Coll RC 16-224 **B***Nanophylliumfrondosum* female Coll RC 16-049 **C***Nanophylliumchitoniscoides*Coll FH**D***Nanophyllium* species NHMUK 012497230.

**Figure 8.**
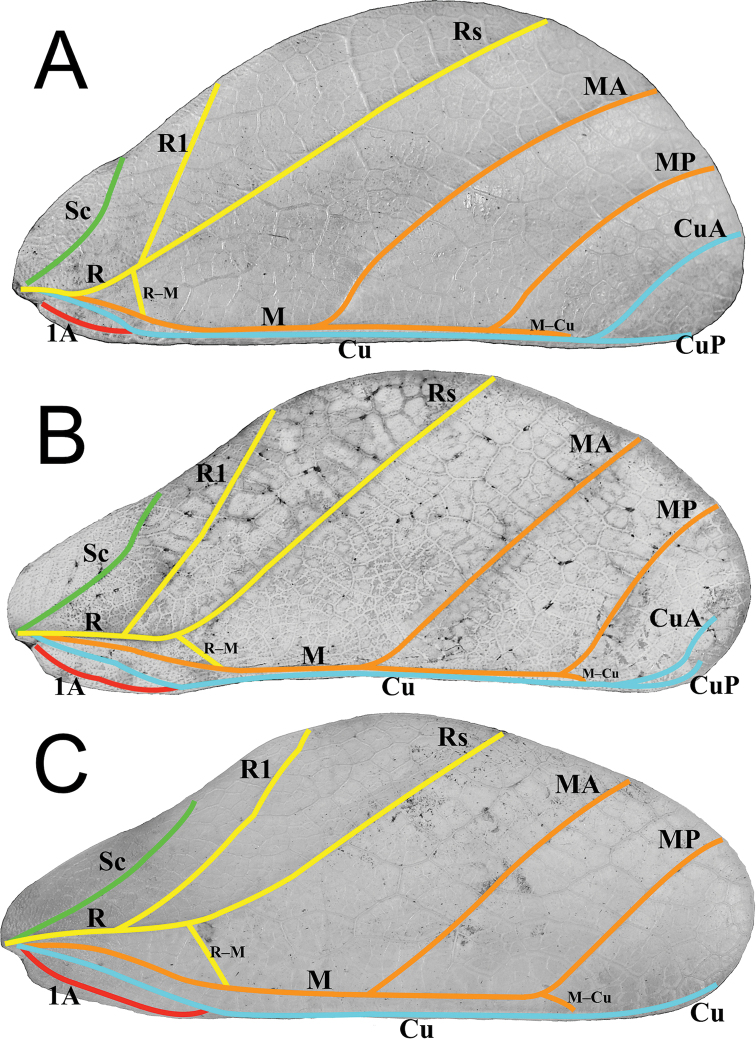
Three primary venation patterns seen in female *Nanophyllium* tegmina, dorsal view **A***Nanophylliumchitoniscoides*Coll FH**B***Nanophylliumsuzukii* holotype, SDEI**C***Nanophylliumkeyicum*Coll FH. Abbreviations used: Sc (subcosta); R (radius); R1 (radius 1); Rs (radial sector); R–M (radius and medial crossvein); M (media); MA (media anterior); MP (media posterior); Cu (cubitus); CuA (cubitus anterior); CuP (cubitus posterior); 1A (first anal).

1. Antennae: consisting of nine or ten segments (Fig. 5).

2. Posteriormedial tubercle of the head capsule, split into two points (Fig. 6A–C) not a single tubercle as is present in most of the remaining Phylliidae (Fig. 6D).

3. Thorax: mesopleurae on their anterior end are notably wider than the prescutum anterior width. Mesopleurae always with prominent tubercles. Prescutum length to width ratio from 1 : 1.8 (more typical) to 1 : 3.4 (in the more extreme width like which is seen in *N.suzukii*) (Fig. 7).

4. Tegmina: within the *Nanophyllium* there are three primary venation patterns (Fig. 8).

a. In the smallest species (individuals ~ 56.0 mm or less), the radial bend occurs before the splitting of the first radial and the radial sector, therefore the radial sector is straight (for example see *N.chitoniscoides* comb. nov.; Fig. 8A). These females also have a radius and medial crossvein present on the radial bend at or before the splitting of the first radial (Fig. 8A). The cubitus at its terminus is clearly split into the anterior cubitus and posterior cubitus veins with a clearly defined gap between them (Fig. 8A). Example species are *N.chitoniscoides* comb. nov., *N.daphne* sp. nov.

b. For the larger species, the bend in the radial vein happens on the radial sector after the splitting of the first radial from the radius (Fig. 8B). Also, the radius and medial crossvein occurs after the splitting of the first radial, instead originating on the radial sector (Fig. 8B). The cubitus at its terminus can be weakly split into the anterior cubitus and posterior cubitus, but in many specimens this vein is simple and unbranched (it is never clearly split with a large gap between the anterior cubitus and posterior cubitus like is seen in the smaller species; Fig. 8A). Example species are *N.asekiense* comb. nov., *N.frondosum* comb. nov., or *N.suzukii* comb. nov.

c. The final venation pattern is found in *Nanophylliumkeyicum* comb. nov., the only species in this genus which has a wide gap between the media and cubitus veins which persists throughout the entire length of the media, this gap is several times wider than a single vein width. This feature is only seen on this species from Kei Island, Indonesia (Fig. 8C). All other examined *Nanophyllium* species have the media and cubitus veins running side by side throughout the entire length, no farther than a single vein width apart (for example in the larger species like *N.asekiense* comb. nov. or *N.suzukii* comb. nov.; Fig. 8B), or with veins moderately wide for the anterior portion (at most two or three vein widths apart) but as they reach the splitting of the media posterior the width between the media and cubitus veins is reduced and the veins are side by side (this example can be seen in the smaller species like *N.daphne* sp. nov.). Also, in *N.keyicum* comb. nov., the radius and medial crossvein occurs after the splitting of the first radial, instead originating on the radial sector (Fig. 8C). Similar to the larger species, the cubitus at its terminus can be weakly split into the anterior cubitus and posterior cubitus (like those seen in Fig. 8B), but in most specimens of *N.keyicum* comb. nov. this vein is simple and unbranched (as illustrated in Fig. 8C).

**Figure 9.**
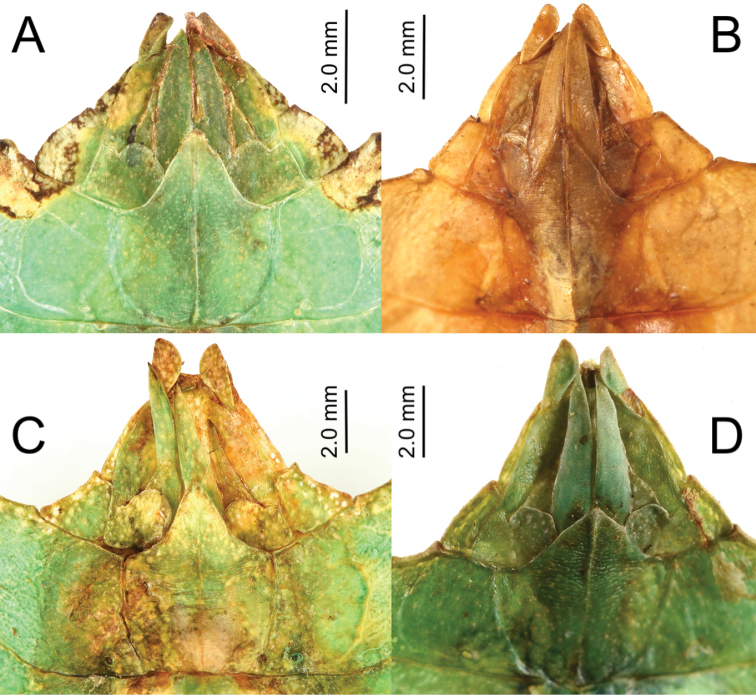
Female *Nanophyllium* genitalia, ventral view **A***Nanophyllium* species NHMUK 012497230 **B***Nanophyllium* species, Papua New Guinea, Central Province, Coll RC 16-224 **C***Nanophyllium* species, Indonesia, West Papua, Mapia, Coll SLT**D***Nanophylliumchitoniscoides*, Coll FH.

5. Alae: absent.

6. Genitalia: gonapophyses protruding from abdominal segment VIII as long as the terminal abdominal segment, gonapophyses protruding from abdominal segment IX thinner and shorter, not exceeding the terminal abdominal segment, and subgenital plate short and moderately broad with the point just reaching the anterior margin of the terminal abdominal segment (Fig. 9).

#### Male *Nanophyllium* general morphology

**Figure 10.**
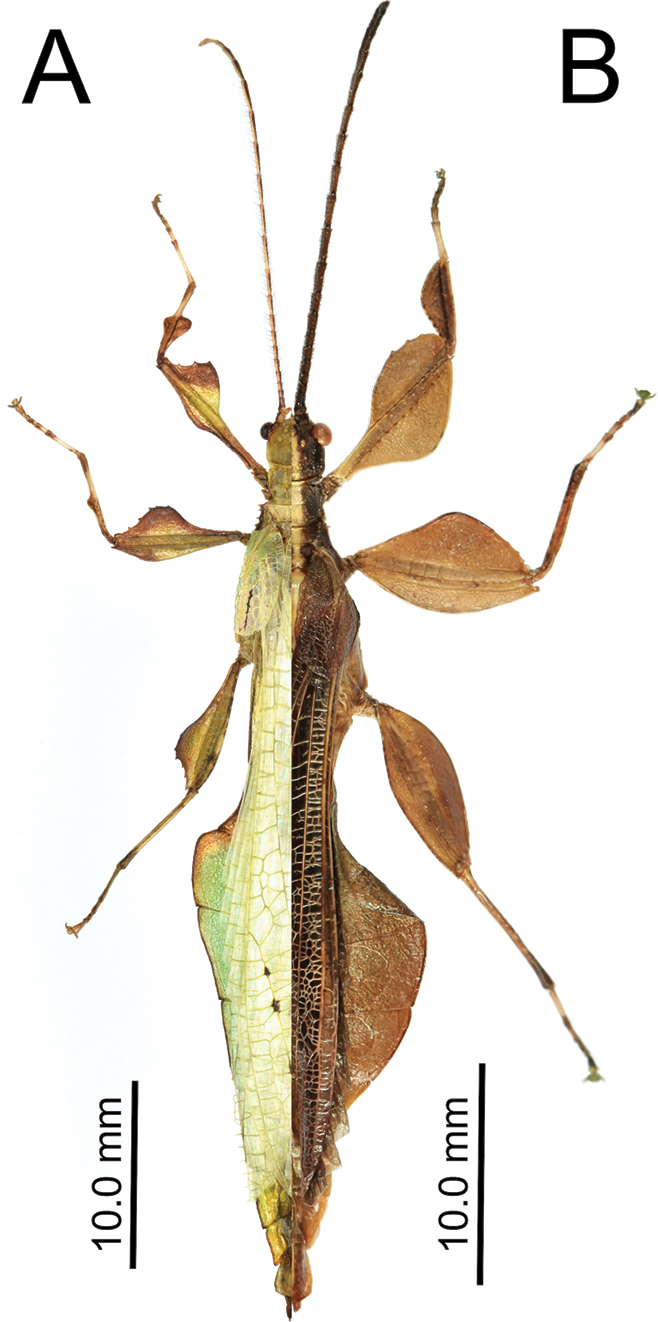
Side by side *Nanophyllium* males of the two species groups **A***pygmaeum* species group, *Nanophylliumasekiense* male from Morobe Province, Papua New Guinea **B***stellae* species group, *Nanophylliumstellae* HT from Jayapura, Indonesia.

The *Nanophyllium* based on male morphology can be separated into two distinct species groups, the *pygmaeum* species group (Fig. 10A) and the *stellae* species group (Fig. 10B). Males of several species for the two species groups are known, but females and eggs are only known for the *pygmaeum* species group, the female and eggs are not yet known for the *stellae* species group.

**Figure 11.**
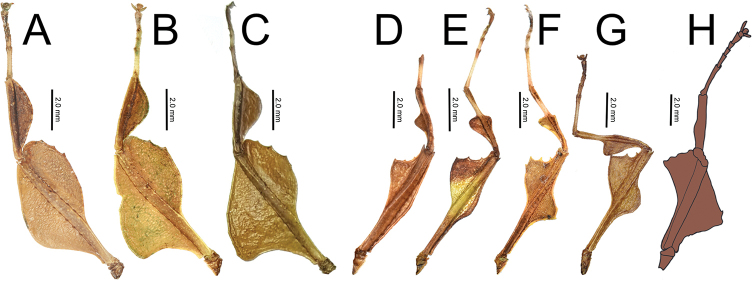
Front legs and lobes of males of the *stellae* species group (**A–C**) and the *pygmaeum* species group (**D–H**) **A***Nanophylliumstellae***B***Nanophylliummiyashitai* sp. nov. **C***Nanophylliumlarssoni***D***Nanophylliumaustralianum***E***Nanophylliumasekiense* comb. nov. **F***Nanophylliumrentzi*Coll SLT**G***Nanophylliumhasenpuschi***H***Nanophylliumadisi* line drawing based on holotype.

Easily observed morphological features which differentiate the species groups are the femoral lobes. The *pygmaeum* species group has profemoral interior lobes which are angular (Fig. 11D–H) and mesofemoral interior lobes which do not reach from end to end of the shaft and have distinct serrate teeth. The *stellae* species group has profemoral interior lobes which are rounded without a sharp angle (Fig. 11A–C) and mesofemoral interior lobes which are a large rounded triangle, reaching from end to end without prominent spination.

**Figure 12.**
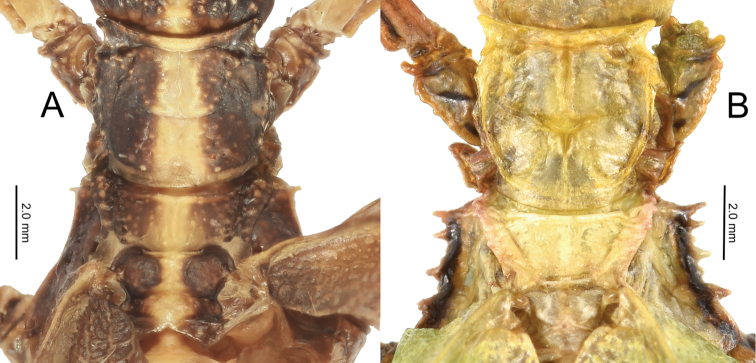
Pronotum and thorax of males for the two species groups, dorsal **A***Nanophylliumstellae* (*stellae* species group) **B***Nanophylliumasekiense* comb. nov. (*pygmaeum* species group).

**Figure 13.**
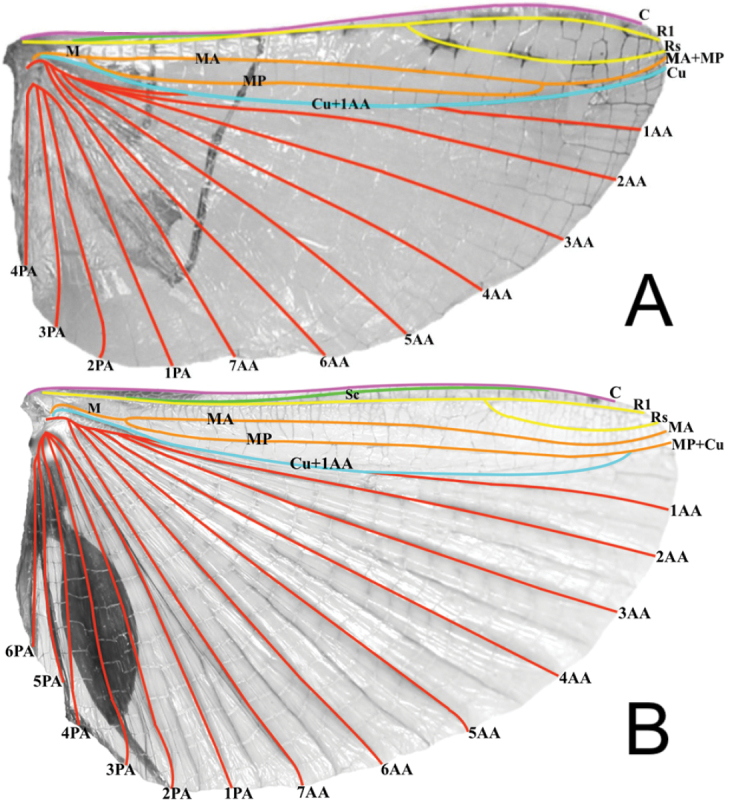
Alae wing venation of males for the two species groups, dorsal **A***Nanophylliumrentzi* (*pygmaeum* species group) **B***Nanophylliumstellae* (*stellae* species group). Abbreviations used: C (costa); Sc (subcosta); R (radius); R1 (radius 1); Rs (radial sector); M (media); MA (media anterior); MP (media posterior); Cu (cubitus); Cu+1AA (cubitus and first anterior anal); 1AA–7AA (first through seventh anterior anal); 1PA–6PA (first through fifth posterior anal).

1. Profemoral interior lobe, in both species groups with most often three small teeth, but occasionally four teeth (rarely), never more than four teeth (Fig. 11A–H).

2. Posteromedial tubercle of the head capsule split into two points (Fig. 7A–C) not a single tubercle as is present in most of the remaining Phylliidae (Fig. 7D).

3. Thorax: mesopleurae on their anterior end are moderately wider than the prescutum anterior width (Fig. 12). Prescutum length to width ratio ranges from 1 : 2.6 to 1 : 3.3 (Fig. 12).

4. Tegmina short, length never exceeding the posterior of the metathorax. The venation of the tegmina appears to be rather simple/too sclerotized to identify details of venation. From a review of several different species it appears as though the subcostal vein is lost within an area that is highly sclerotized. The radial vein is moderately present and runs along the edge of the highly sclerotized patch. The medial vein is the most prominent and runs through the center of the tegmina and occasionally has a weak vein splitting from it, but for most observed specimens the medial vein was not prominently branched. The cubitus and first anal are only moderately formed and give stability to the other half of the tegmina and are not notably branched.

5. A unique feature of the alae which appears to be a symplesiomorphy for the *Nanophyllium* is that the radius splits into the first radial and the radial sector on the distal half of the wing and these two veins run separately to the wing margin without fusing to others (the only other group which also has the radial split on the distal half is the *Pulchriphyllium*, but their radial sector fuses with the media anterior, media posterior, and the cubitus instead of running by itself to the wing margin; all other leaf insect genera have the radial split happening on the proximal half of the wing). Within the *Nanophyllium* the alae venation differs between the two species groups.

a. In the *pygmaeum* species group the key differences are that the media posterior fuses back to the media anterior before reaching the wing margin, and then the fused media runs on its own to the wing margin without fusing with the radial sector (Fig. 13A).

b. In the *stellae* species group the media anterior and the media posterior do not fuse, instead they both run to the wing margin, and the cubitus after splitting from the first anterior anal fuses with the media posterior near the wing margin and then they run fused to the margin as one (Fig. 13B).

6. Vomer long and slender with a single apical hook (Fig. 19C).

#### Nanophyllium egg general morphology

Figure 14

Fortunately, there are several *Nanophyllium* species with the egg morphology known.

**Figure 14.**
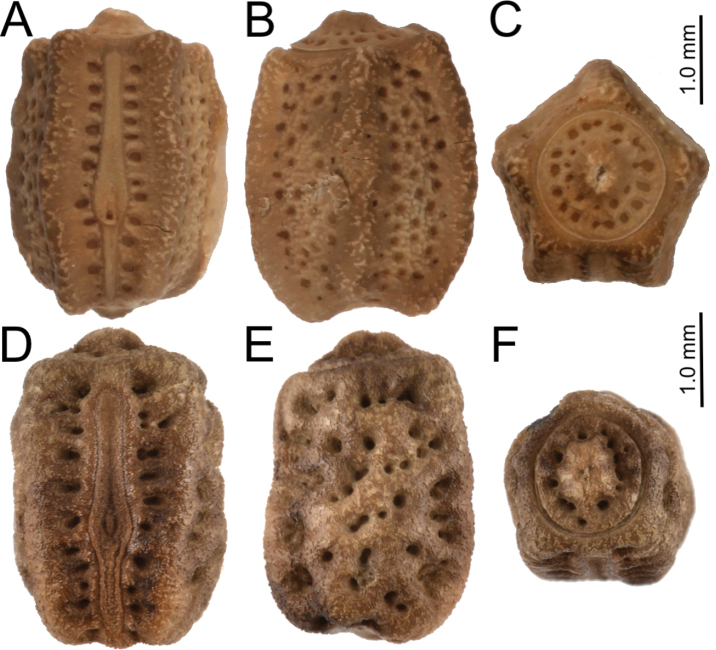
**A–C***Nanophylliumasekiense*Coll RC 18-046 **A** dorsal view **B** lateral view **C** Opercular (anterior) view **D–F** egg from *Nanophyllium* sp. NHMUK 012497230 **D** dorsal view **E** lateral view **F** opercular (anterior) view.

These species include: *Nanophylliumasekiense* (Figs 3, 14A–C) comb. nov.; *Nanophylliumchitoniscoides* (Größer, 1992: 165, fig. 3), comb. nov.; *Nanophylliumkeyicum* (Karny, 1914: see Größer, 2008: 123, fig. 146), comb. nov.; *Nanophylliumsuzukii* (Größer, 2008: 137, fig. 171), comb. nov.; and the unidentified *Nanophyllium* female from the NHMUK 012497230, has a single egg mounted to a card below the specimen (Fig. 14D–F). From these known eggs a generalized list of morphological features can be compiled.

1. Cross-section is roundly pentagonal (Fig. 14C, F).

2. Surface is roughly textured, with pitting of various sizes throughout the capsule surface. Surface lacks pinnae. Pits on the capsule surface depending on the species can either have significant depth to them or in other species can have shallow pits.

3. Operculum has distinct pitting surrounding the central gently raised apex (Fig. 14C, F).

4. Micropylar plate is elongate, nearly reaching from end to end of the capsule and with an approximately uniform width throughout except for around the micropylar cup where it is slightly wider. Running parallel along the micropylar plate margin are pits, which vary in number from species to species (Fig. 14A, D).

5. Lateral surface with irregular pitting in no detectable pattern, with some pits very near each other or touching to form wider irregular shapes (Fig. 14B, E).

#### *Nanophyllium* Distribution

Figures 4, 15

**Figure 15.**
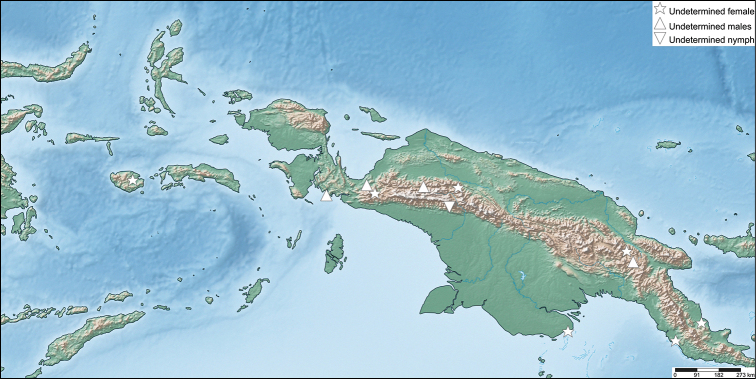
Distribution map showing the undetermined *Nanophyllium* specimens with mappable localities.

INDONESIA

**Figure 16.**
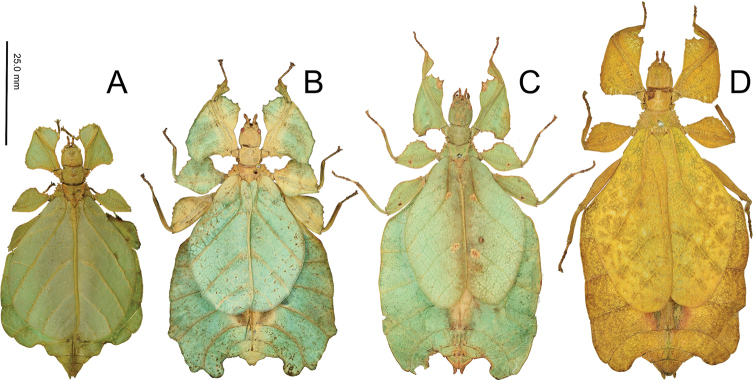
Notable known *Nanophyllium* females scaled to relative size **A***Nanophylliumchitoniscoides* comb. nov. Coll FH**B***Nanophylliumsuzukii* comb. nov. **C***Nanophylliumfrondosum* comb. nov. **D***Nanophylliumkeyicum* comb. nov. (RMNH).

North Maluku Province:

Batjan Island (*N.suzukii*: SDEI, Fig. 16B)

Maluku Province:

Buru Island (*Nanophyllium* sp. Undetermined female)

Kei Island (*Nanophylliumkeyicum*: NHMUK; RMNH; SDEI; FH Coll, Fig. 16D)

West Papua Province:

Fak Fak (*Nanophylliumrentzi*: NHMUK, Fig. 18A; Coll SLT, Fig. 18B)

Aiduma Island (*Nanophyllium* sp. Undetermined male, Fig. 22)

Papua Province:

Biak Numfor Regency, Biak Island (*N.daphne* sp. nov., RMNH, Fig. 21C)

Nabire Regency (*N.hasenpuschi*: NHMUK)

Dogiyai Regency, Mapia (*Nanophyllium* sp. Undetermined female: Coll SLT, Fig. 24F)

Mimika Regency, Utakwa River (*Nanophyllium* sp. Undetermined male, Wollaston Expedition, NHMUK)

Nduga Regency “Hoofdbivak” (*N.adisi*, Stirling Expedition, SMTD)

Nduga Regency “Kloofbivak” (Third South New Guinea Expedition, *Nanophyllium* sp. Undetermined nymph, ANSP)

Central Mamberamo Regency, Kobakma (*Nanophyllium* sp. Undetermined female, CAS, Fig. 24E)

Jayapura Regency, Cyclops Mts. (*N.stellae* (Fig. 10B and *N.larssoni*, SDNHM)

PAPUA NEW GUINEA

Western Province:

Katau (*N.pygmaeum*: MSNG)

Daru Island (*Nanophyllium* sp. Undetermined female, CAS, Fig. 24D)

Chimbu Province:

Kerowagi District (*N.frondosum*, Coll RC, Fig. 16C)

Eastern Highlands Province:

Mt. Otto (*Nanophyllium* sp. Undetermined female nymph, Sixth Archbold Expedition, AMNH, Fig. 24A)

Buntibasa dist., N. Guinea, Kratke Mts, 4,000–5,000’, February 1933. (F. Shaw Mayer), (*Nanophyllium* sp. Undetermined male nymph, NHMUK)

Herowana Village (observational record for *N.asekiense* by Daniel Levitis, USA)

Gulf Province:

Kerema (*N.asekiense*, Coll RC)

Morobe Province:

Wau (*N.miyashitai* sp. nov., IMQC, Fig. 20)

Lae (*N.asekiense*, Coll RC)

Sattelberg (*N.frondosum*, UMMZ)

Menyama District, Aseki (*N.frondosum* and *N.asekiense*, Coll RC; *N.chitoniscoides*, Miyashita Private collection, and Coll FH Fig. 16A)

Watut (*N.chitoniscoides*, paratype in the Detlef Größer private collection)

Central Province:

Vanama River (*Nanophyllium* sp. Undetermined female, Coll RC, Fig. 24B)

Port Moresby (*Nanophyllium* sp. Undetermined female, NHMUK, Fig. 24C)

Northern Province:

Popondetta (*Nanophyllium* sp. Undetermined female, NHMUK)

Milne Bay Province:

Normanby Island (*Nanophyllium* sp. Undetermined female)

AUSTRALIA

Queensland:

Iron Range National Park (*N.australianum*, ANIC)

Lockhart (*N.australianum*, Fig. 23)

##### Nanophyllium asekiense

Taxon classificationAnimaliaPhasmidaPhylliidae

(Größer, 2002) comb. nov.

5779D91D-4D07-5D0E-A363-A566F894971C

Figures 1 , 2 , 3 , 6A , 10A , 11E , 12B , 14A–C , 17 , 19

###### Discussion.

Female *N.asekiense* comb. nov. is most often confused with the sympatric species *N.frondosum* comb. nov., but *N.asekiense* comb. nov. can be differentiated by the presence of small exterior pro- and meso-tibial spurs, which *N.frondosum* comb. nov. lacks.

**Figure 17.**
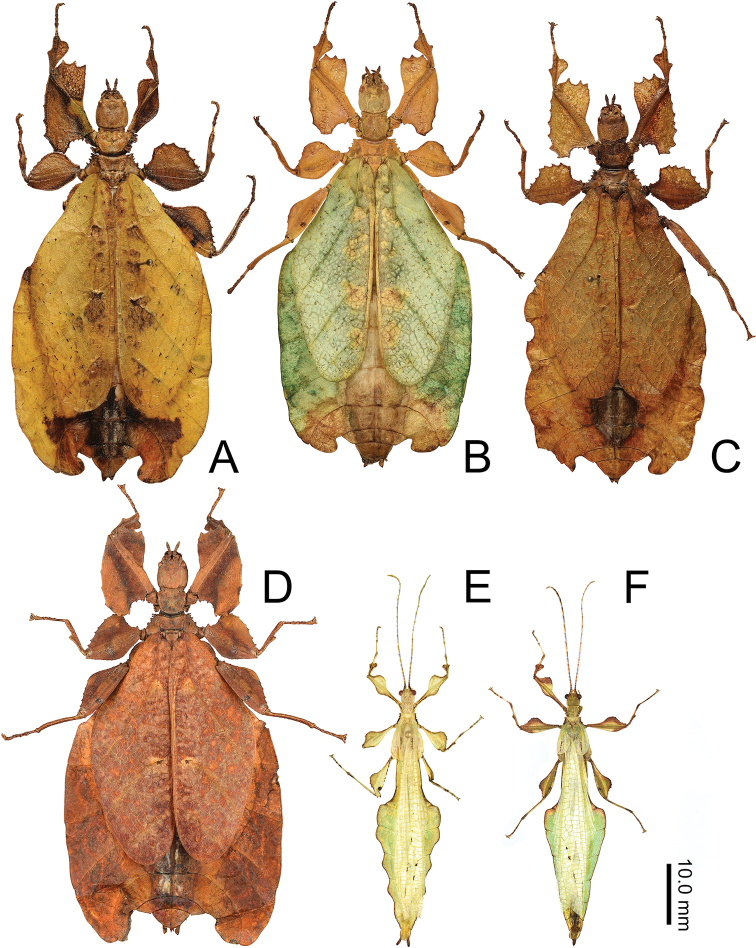
*Nanophylliumasekiense* (Größer, 2002), comb. nov. males and females, all originating from Papua New Guinea, Morobe Province. Note the variation in the male abdominal shape and the female abdominal, color, and femoral lobe variability **A** yellow form female, Morobe Province, Aseki, Oiwa Village, July, 2016, Coll RC 16-268 **B** green form female, Papua New Guinea, Morobe, Aseki (Oiwa), Nov. 2000, Coll SLT**C** brown form female, Morobe Province, Aseki, Oiwa Village, July, 2016, Coll RC 16-264 **D** red/brown form female, Papua New Guinea, Morobe, Aseki (Oiwa), Nov. 2000, Coll SLT**E** serrate abdominal male bred by the Montreal Insectarium, IMQC**F** spade shaped male bred by the Montreal Insectarium, Coll RC 19-055.

**Figure 18.**
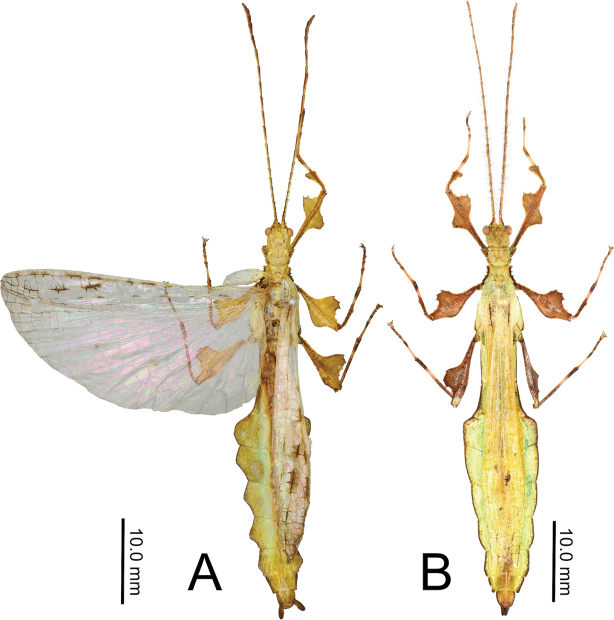
*Nanophylliumrentzi* males dorsal, note the variation in abdominal shape **A** holotype in the NHMUK (copyright NHMUK 2020 online Data Portal; https://data.nhm.ac.uk/object/b03f481f-1a1c-4ccd-8558-505668fc78f3/1591228800000) **B** male from Fak Fak, Indonesia, Coll SLT.

Only two male *N.asekiense* comb. nov. are known at present, but their morphology is consistent between them for all features except for the abdominal shape (see Fig. 17E, F for side by side comparison of these two males). An additional specimen of *Nanophylliumrentzi* also shows how variable male abdominal morphology can be within the same species while the other morphological features remain stable (Fig. 18A, B). *Nanophylliumasekiense* comb. nov. males can be differentiated from the other known *Nanophyllium* males based on the profemoral lobe morphology. In *N.asekiense* comb. nov. the interior profemoral lobe is distinctly right-angled (a feature present in all species of the *pygmaeum* species group (Fig. 11E–H) except *N.australianum* which has a thinner, obtuse angle; Fig. 11D), but the exterior profemoral lobe of *N.asekiense* comb. nov. is narrow, only about the same width as the profemoral shaft; Fig. 11E (more like *N.australianum* than any other species, as the other species instead have an exterior profemoral lobe which is broader than the width of the profemoral shaft; Fig. 11F–H).

###### Description.

**Male. *Coloration*.** Each antennal segment with dark brown and tan coloration. The rest of the body and legs are of a yellow-green based color, with variable brown margins. In the two males bred by the Montreal Insectarium the individual with the undulating abdomen has minimal brown markings, with only brown along the leg margins and the abdomen margins (Fig. 17E). The male with the straight abdominal margins has more prominent brown markings throughout, with around half of the leg lobes marked with brown and a wider brown abdominal margin (Fig. 17F). The alae and tegmina on both males are a translucent pale green, with small flecks of dark brown along the prominent veins.

**Figure 19.**
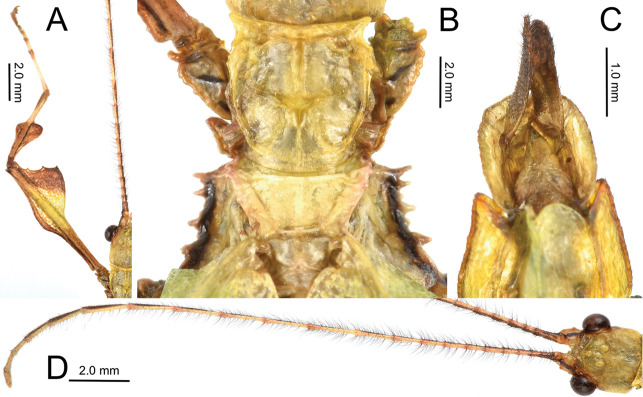
Male *Nanophylliumasekiense* (Größer, 2002), comb. nov. morphological details **A** profemoral and protibial lobes **B** dorsal thorax spination **C** ventral view of the genitalia **D** dorsal view of the head and antennae.

***Morphology*.***Head*. Head capsule about as long as wide, with a vertex that is lumpy without notable granulation, two posteromedial tubercles are not notably large but are present with a slight furrow between them (Fig. 19D). Compound eyes are notably protruding from the head capsule and there are three well-developed ocelli between and slightly posterior to them (Fig. 19D). *Antennae*. The antennae are longer than the outstretched forelegs and consist of 23 antennomeres (Fig. 19D). The scapus and pedicellus are nearly bare, with only a few short clear setae. All segments beyond the scapus and pedicellus except for the terminal four are covered in stiff dark setae which are each longer than the segment they are on is wide. The terminal four antennal segments also have dark setae, but these setae are shorter than the segments are wide (Fig. 19D). *Thorax*. Pronotum as wide as it is long with moderately formed rims on the lateral margins that are mostly parallel and only gently converging near the posterior, the anterior margin is slightly curved with a prominent rim, and the posterior margin is weakly formed (Fig. 19B). Surface of the pronotum has a distinct sagittal furrow and central lateral furrow, and the surface is irregularly lumpy, but not granular (Fig. 19B). Prescutum significantly wider than long, at its widest on the anterior it is 2.5 times wider than long. The prescutum margins evenly converge toward the posterior and have two or three notable nodes on the anterior portion, and the remainder of the margin is irregularly lumpy. Surface of prescutum is nearly smooth without significant features, and the anterior prescutum margin is simple, also lacking nodes or spines (Fig. 19B). Mesopleurae gradually diverging wider from anterior to the posterior, and marked with irregularly shaped tubercles throughout, with around four notably larger ones and smaller nodes intermixed (Fig. 19B). Mesopleurae surface irregularly lumpy with a single distinct pit in the center and no notable nodes. Pro-, meso-, and meta-sternum with irregularly spaced granules mostly along the sagittal plane but with the margins occasionally with sparse granules. *Wings*. Tegmina short, not exceeding the posterior of the metathorax. The subcostal vein and any splitting of the radius is obscured within an area of the wing that is highly sclerotized. The radius is the most prominent vein and runs through the center of the tegmina to the posterior margin (along the edge of the highly sclerotized patch). The medial vein is also prominent and runs through the center of the tegmina parallel with the radius and does not appear as though the media has any notable splits. The cubitus and first anal are moderately formed and give stability to the other half of the tegmina, are not notably branched, and this half of the tegmina is not as heavily sclerotized as the other half. The first anal fuses with the cubitus around two thirds of the length, and the cubitus runs nearly to the wing margin. Alae developed, with the exposed section of folded alae slightly sclerotized, but not as sclerotized as the alae. The costa runs along the wing margin with a weak subcosta running along and eventually fusing with it. The radius splits into the first radial and the radial sector just distal to the wing midline and these two veins run separately to the wing margin without fusing to others. The media splits into the media anterior and the media posterior almost immediately near the base of the wing. The media posterior fuses back to the media anterior near the distal one fifth of the wing, and then the fused medial veins run to the wing margin (Fig. 13A). The cubitus runs with the first anterior anal for most of the length and then near the distal one fifth of the wing they split and the cubius runs unbranched and unfused to the wing margin. There are seven anterior anals which run simply to the wing margin and four or five well-formed posterior anals which run simply to the wing margin. *Abdomen*. Abdominal segment II with parallel sides, segment III widening in a smooth arc, segment IV widening slightly for the first half, then gently curving in for the second half, segments V through VIII variable with margins that are either straight and converging uniformly to the apex which gives the abdomen a straight spade-shaped appearance or with margins which each expand and then contract which gives the abdomen a lobed appearance. Abdominal segment IX with margins which slightly converge to the abdominal segment X which is longer than wide and ends in a rounded apex. *Genitalia*. Poculum broad and ending in a slightly cleft apex that reaches the anterior margin of segment X (Fig. 19C). Cerci densely covered in nodes throughout the surface and short setae mostly on the distal half (Fig. 19C). Vomer long, and slightly bending to the side, not perfectly straight; with sides gradually converging to the upward hooked apex (Fig. 19C). *Legs*. Profemoral interior lobe angular (approximately 90 degrees) with three small evenly spaced teeth with a nearly straight gap between each tooth (Fig. 19A). Exterior profemoral lobe significantly thinner than the interior lobe (about as wide as the profemoral shaft), only present as a smoothly curved lobe just distal to the midline, not spanning the entire length (Fig. 19A). Protibiae with lobes only present on the proximal half, the distal half is bare. The protibial exterior lobe is a scalene triangle only as wide as the protibial shaft. The interior lobe is also a scalene triangle and about two times as wide as the exterior lobe (Fig. 19A). Mesofemoral exterior lobe smoothly arcing the full length, with the widest portion on the distal half and only about one and a half times as wide as the mesofemoral shaft, with fine serration on the widest portion only. Mesofemoral interior lobe with the majority of the lobe on the distal half, the proximal half with only a sliver of the lobe and lacks teeth versus the wide proximal expanse (about three times as wide as the mesofemoral shaft) has four to five notable serrate teeth. Interior metafemoral lobe with the majority of the lobe on the distal half, with only a thin sliver on the proximal half. The distal half of the metafemoral lobe is two and a half times as wide as the shaft and has five to six serrated teeth. Exterior metafemoral lobe slightly thinner than the metafemoral shaft, spanning the full length, and lacking serration. Mesotibial exterior has a small roundly triangular lobe near the midline which is only about as wide as the mesotibial shaft. Metatibiae lacking exterior and interior lobes.

##### Nanophyllium miyashitai sp. nov.

Taxon classificationAnimaliaPhasmidaPhylliidae

D7798876-1147-5C8F-98AE-1232313315AD

http://zoobank.org/2C3C5029-9E78-4687-A680-BDC9645DD3CB

Figure 20

###### Type material.

***Holotype***: ♂, Papua New Guinea: Morobe Province, Wau: IX, 2000. From the collection of Tetsuo Miyashita, Japan. Deposited in the Montreal Insectarium (Quebec, Canada) type collection.

###### Differentiation.

With the interior lobe of the profemora rounded, not angular, and the mesofemoral interior lobe broad and reaching fully end to end in a rounded triangle, this new species falls within the *stellae* species group. This is the first species from the *stellae* species group recorded from Papua New Guinea. The other two species are known from Jayapura, Irian Jaya, Indonesia (very near the border with Papua New Guinea so it is likely they also occur there but to date, we have not confirmed any specimens). This new species can be differentiated from the other two species in the *stellae* species group by the mesopleurae which have a prominent anterior tubercle followed by four additional small tubercles (only a single anterior tubercle in the other two species) and tegmina that are shorter, only about half the length of the metathorax (almost the length of the metathorax in the other two species).

Like the other members of the *Nanophylliumstellae* species group, the holotype is a male specimen and the female is unknown. It is expected that the female is larger than other known female *Nanophyllium* as the *stellae* species group members are larger than the *pygmaeum* species group members.

###### Description.

**Male. *Coloration*.** Antennae dark brown, a similar brown to that found throughout the head and thorax. The majority of the dorsal aspect throughout the remainder of the body and legs is of a similar lighter brown, but not a light as the stripe of light brown running along the sagittal plane along the head and thorax. Alae and tegmina have a similar dark brown to that found on the antennae. Throughout the ventral surface the coloration is the same as that found on the legs. Granulation on the body is mostly of a lighter brown than the surface it is found on.

**Figure 20.**
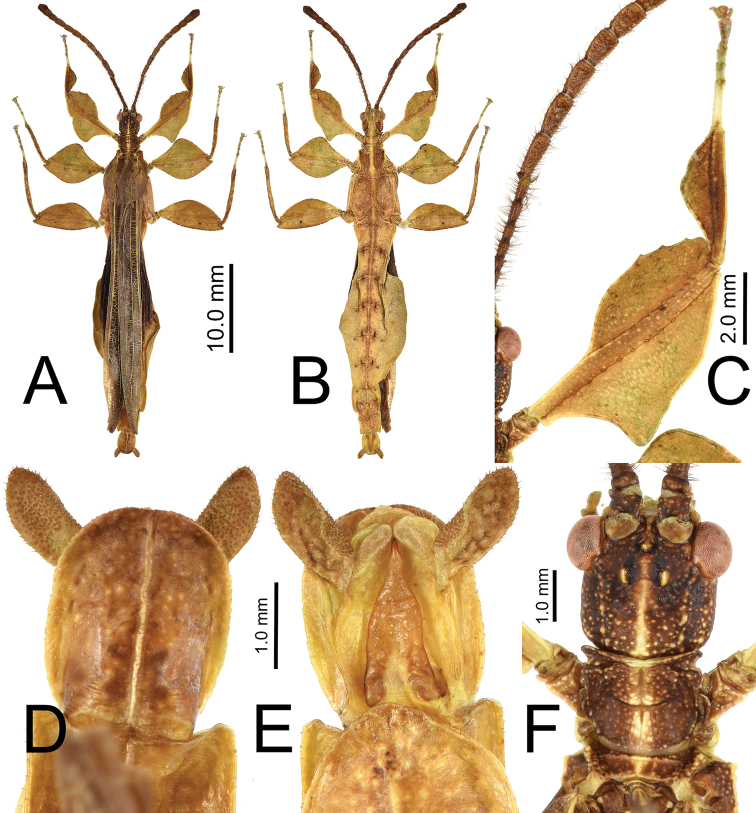
Holotype male *Nanophylliummiyashitai* sp. nov. **A** full body dorsal **B** full body ventral **C** right front leg **D** abdominal segment X and dorsal view of cerci **E** genitalia, ventral **F** head through thorax, dorsal.

***Morphology*.***Head*. Head capsule slightly longer than wide, with a vertex that is heavily granulose, which includes the two posteromedian tubercles which are no larger than the surrounding granulation around them (Fig. 20F). Three well-developed ocelli are slightly posterior to the compound eyes which are ovular and slightly protrude from the head capsule (Fig. 20F). *Antennae*. Antennae in the holotype are both damaged and repaired so the original number of antennomeres is unknown. The antennae are longer than the outstretched forelegs and the left antennae consist of 21 antennomeres and the right of 19 (including the scapus and pedicellus). Scapus and pedicellus with short clear setae and the scapus has a notable spur on the anterior rim lateral side. All segments beyond the scapus and pedicellus covered in stiff dark setae each longer than the segment is wide until the terminal four segments where the setae begin to steadily decrease in size until the terminal segment which has dense short setae. *Thorax*. Pronotum wider than long (width to length, 1 : 0.75) with parallel lateral margins, and all margins slightly granulose. Surface of the pronotum heavily granulose like the vertex of the head capsule. Prescutum significantly wider than long (width to length, 3.3 : 1), with converging lateral margins with a granular surface of at least five nodes (Fig. 20F). Surface of prescutum slightly granular but lacking significant features. Mesopleurae gently diverging, anterior edge armed with a single tubercle, remainder of the rim with four small tubercles with a single seta protruding from the tip of each. Mesopleurae surface irregularly granular with a single distinct pit in the center. Pro-, meso-, and metasternum covered in irregularly spaced granules. *Wings*. Tegmina short, only reaching about halfway through the metathorax. Alae developed; exposed section of folded alae moderately sclerotized. *Abdomen*. Abdominal segments with folding in the holotype so shape description is only approximate. Abdominal segment II slightly tapering, III gradually widening, IV widening for the first quarter, then parallel, V through the first half of VI parallel, VII converging, VIII–IX parallel to subparallel. Anal abdominal segment X longer than wide with a broad rounded apex (Fig. 20D). Poculum broad, about as broad as segment IX, ending in a broad rounded apex that reaches the anterior margin of segment X (Fig. 20E). Cercus about as wide as the vomer but slightly shorter, margins marked with a row of thin tan setae and a dorsal surface that is heavily granular. Vomer long, reaching the majority of the length to the apex, with sides gradually converging to the hooked apex (Fig. 20E). *Legs*. Profemora, interior lobe rounded with three small nubby, evenly spaced teeth (Fig. 20C). Exterior lobe wider than the interior lobe and with a slight recurve and an edge that is smooth with a row of single stout setae along the entire length. Protibiae lacking an exterior lobe, interior lobe a rounded scalene triangle spanning the entire length of the protibia (Fig. 20C). Mesofemora, exterior lobe smoothly arcing the length of the mesofemora, interior lobe smoothly triangular with five to six small nubby teeth on the distal half and about one and a half times as wide as exterior lobe. Interior and exterior lobe of metafemora smoothly arcing with interior lobe about twice as wide as the exterior lobe and the interior with a few small nubby teeth near the distal end. Meso- and metatibiae lacking exterior and interior lobes.

###### Measurements of holotype

[**mm].** Length of body (including cerci and head, excluding antennae) 40.0, length/width of head 2.6/2.5, antennae (repaired) 16.4, pronotum 1.8, mesonotum 1.8, length of tegmina 5.9, length of alae 30.5, greatest width of abdomen 8.0, profemora 7.4, mesofemora 6.8, metafemora 7.3, protibiae 3.9, mesotibiae 5.0, metatibiae 6.7.

###### Distribution.

Currently only known from the type locality of Wau, Morobe Province, Papua New Guinea (Fig. 4).

###### Etymology.

Patronym. This species is dedicated to Mr. Tetsuo Miyashita (Japan). Miyashita is a major private collector who has amassed one of the largest insect collections in the world. Miyashita and the specimens from his collection have allowed the description of several new beetle taxa over the years with this being the first phasmid described from his collection.

##### Nanophyllium daphne sp. nov.

Taxon classificationAnimaliaPhasmidaPhylliidae

BFB9DCC4-8E59-5CEC-B91D-50D00C3056F5

http://zoobank.org/A5CB1FE9-AA0A-4141-A291-C20654161E50

Figure 21

###### Type material.

***Holotype*** ♀: Indonesia: Biak. 16/9.54; NNM-Leiden, ex collectie A. Veldhuyzen. In the collection RMNH, Leiden, Netherlands.

###### Discussion and differentiation.

This small species (body length of the holotype only 54.0 mm) has several interesting morphological features which differentiate it from other known *Nanophyllium* females. The tegmina venation places this species most closely aligned to *N.chitoniscoides* due to the venation pattern having the radial bend occurring before the splitting of the first radial and the radial sector, therefore the radial sector is straight (Fig. 9A). Additionally, a radial and medial crossvein is present on the radial bend at or before the splitting of the first radial (Fig. 9A).

**Figure 21.**
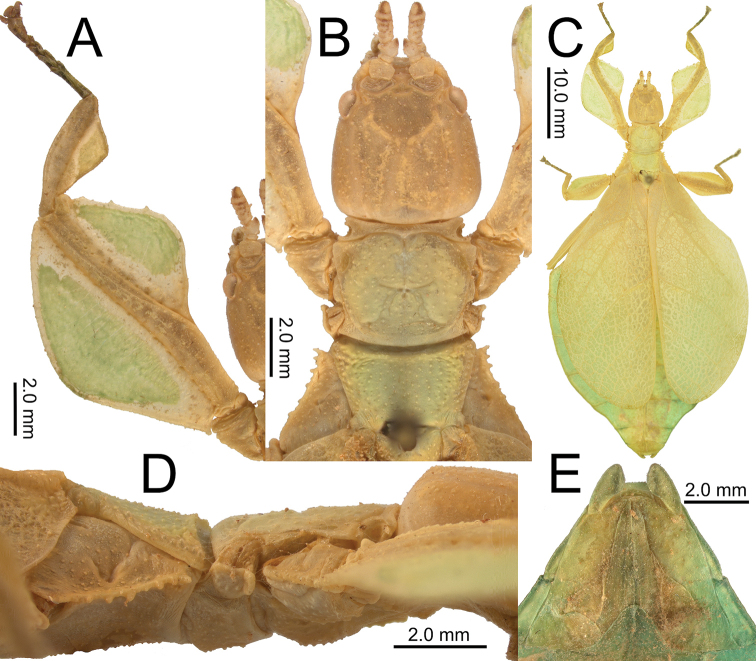
Female holotype of *Nanophylliumdaphne* sp. nov. **A** front left leg showing lobes and serration **B** antennae, head, and thorax dorsal details **C** full body dorsal **D** thorax, lateral view **E** genitalia, ventral.

This new species can be differentiated from all other *Nanophyllium* by several morphological features. First, it is the only species which has exterior profemoral lobes which are obtuse (Fig. 21A), not right angles like in *N.keyicum* (Fig. 16D) or recurved acute angles like in all other known *Nanophyllium* species (for example Fig. 16C). Additionally, this is the only species known where the female has the abdomen tapering towards the posterior, giving the abdomen a spade-shaped appearance (Fig. 21C), all other known species have females with abdominal segments VI and VII either parallel sided (like in *N.frondosum* and *N.keyicum*, Fig. 16C, D respectively) or as the broadest segments (like in *N.chitoniscoides* and *N.suzukii*, Fig. 16A, B, respectively).

These unique morphological features coupled with the geographic isolation from the mainland makes it unlikely that this female represents the unknown female sex of one of the many species which are only known from males from the mainland (Fig. 4). Instead, we here describe this species as *Nanophylliumdaphne* sp. nov. as the first recorded Phylliidae species from Biak Island, Indonesia.

###### Description.

**Female. *Coloration*.** Presently, only the dried holotype specimen is known, which is fairly well-preserved with only minimal discoloration along the midline due to a lack of gutting. The majority of the body is of a pale light green coloration, with the areas of discoloration (such as the head, thorax, and shafts of the legs) being a pale brown/tan in coloration. Leaf insects are more vibrantly colored in life and it can be assumed that this specimen was a brighter green in life.

***Morphology*.***Head*. Head capsule slightly longer than wide, vertex with small granulation throughout the surface and unevenly spaced in no detectable pattern (some right next to each other some with more spacing). The posteromedial tubercle is small, only slightly noticeable and split into two lobes. Frontal convexity stout, not prominently protruding, with a lumpy surface which is marked by numerous pale setae. *Antennae*. Antennae consisting of nine segments. The terminal segment has a narrower base than segment VIII, instead with a width only about as wide as segments IV or V, and it is about as long as the previous two segments combined length. All segments have setae present; segments I through III have sparse but long pale setae; segments IV through VIII have sparse, stout, tan setae; and the terminal segment IX has dense, stout, dark setae. Compound eyes slender and tightly formed to the head, only reaching across one quarter of the head capsule length. Ocelli absent. Antennal fields approximately the same dimensions as the compound eyes, wider than the base of the first antennomere, and not protruding back farther than the frontal suture. *Thorax*. Pronotum with anterior margin that is slightly concave and lateral margins that are straight that slightly converge to a broad, slightly convex posterior margin that is about the same width as the anterior rim (Fig. 21B). The pronotum surface has moderate granulation throughout that is evenly spaced, and the pronotum surface has a moderate pit in the center and furrows along the sagittal and lateral planes (Fig. 21B). Pronotum lacks prominent rims, with only the anterior rim moderately formed and with a rough texture (but no features as prominent as actual granulation present). Pro-, meso-, and metasternum with granulation throughout, with all granules evenly spaced and of even size. Prescutum wider than long, with an anterior margin 1.3 times wider than the posterior margin (Fig. 21B). Prescutum lateral rims and surface of the prescutum with granulation throughout, but no prominent spination. No prescutum crest present, the surface is only slightly raised so it is not perfectly flat, but it is not prominent. Prescutum anterior rim slightly raised in the center but not prominent, and lacks a sagittal spine, instead there is only weak granulation throughout the rim which is similar to the granulation found on the prescutum surface. Mesopleurae start near the anterior margin but not flush with it, instead they begin notably wider than the prescutum anterior margin. Mesopleurae are nearly straight and diverge evenly along their length (Fig. 21B). Mesopleurae margins on their anterior margin are marked by a prominent tubercle immediately adjacent to two more which are medium sized and followed by three small tubercles that are nearly evenly spaced throughout the remainder of the length of the mesopleurae with slight granulation interspersed (Fig. 21B). Face of the mesopleurae has a granular surface similar to the texture of the prescutum disk and marked with a distinct pit near the middle of the surface. *Wings*. Tegmina long, reaching past the anterior margin of abdominal segment VIII. The subcosta (Sc) is the first vein in the forewing and arcs smoothly unbranched towards the wing margin. The radius (R) gently bends towards the wing margin almost immediately and along this bend (first on the medial side) there is a notable radius to media crossvein (R-M), then following this first branching, the radius branches (on the distal side) into the first radius (R1) which runs unbranched to the wing margin, and the remainder of the radius as the radial sector (Rs) runs unbent to the wing margin, terminating slightly past the wings mid-length. The media (M) runs nearly parallel with the cubitus along the wing margin (there is a slightly wider than side by side gap near the anterior, but the veins are almost touching throughout a majority of their length). The media anterior (MA) diverges near the wing mid-length and arcs smoothly towards the wing margin where it terminates approximately three-quarters of the way through the length of the wing; this is followed by a splitting of the media posterior (MP) which runs parallel with the media anterior as it smoothly arcs towards the wing posterior margin. Following the media posterior split there is a small media to cubitus crossvein (M-Cu) which runs briefly parallel side by side with and then fuses to the cubitus. The cubitus (Cu) is bifurcate, branching into the cubitus anterior (CuA) and cubitus posterior (CuP) which diverge evenly, and both terminate at or near the wing posterior apex. The first anal vein (1A) is simple and fuses with the cubitus near the wing anterior margin. Alae rudimentary. *Abdomen*. Abdominal segments II through the anterior one third of IV uniformly diverging, posterior two thirds of IV through the anterior half of V parallel, the remainder of the abdominal segments are roundly converging to the broad rounded apex giving the abdomen an overall rounded appearance. *Genitalia*. Subgenital plate short and rounded, starting at the anterior margin of segment VIII and extending only about halfway onto segment IX, with straight, uniformly converging margins. Subgenital plate is only about a third the length of the gonapophyses, leaving a significant amount of the gonapophyses exposed. Gonapophyses are long and slender, not quite reaching the apex of the terminal abdominal segment (Fig. 21E). Cerci broad and slightly cupped, with a surface throughout that is rough in texture, and margins with only a few short setae, none prominent. *Legs*. Profemoral exterior lobe broad with a rounded obtuse angle, and slightly wider than the interior lobe. Edge of the profemoral exterior lobe without notable teeth but with a margin that is granular throughout the length (Fig. 21A). Profemoral interior lobe narrower than the exterior and shaped as a slightly obtuse angle marked with four small teeth (Fig. 21A). The proximal most tooth is very small, not much more than a bump along the margin, this is followed by a narrow gap, the first prominent tooth, then a larger gap twice as wide as the first, another prominent tooth the same size as the previous, a gap the same size as the first small gap, and then one more prominent tooth at the distal end which is about the same size as the previous two teeth. The gaps between teeth are not deep and looping, instead they are straight and shallow between each tooth (Fig. 21A). Mesofemoral exterior lobe arcs smoothly from end to end and lacks dentition. The interior and exterior mesofemoral lobes are of a similar width. Mesofemoral interior lobe arcs end to end with three serrate teeth only on the distal quarter of the lobe, which is slightly wider than the proximal portion of the lobe. Metafemoral interior lobe arcs end to end with the distal end wider than the proximal, and seven to eight irregularly shaped teeth on the distal third of the lobe only. Metafemoral exterior lobe is thin, smooth, and hugs the metafemoral shaft without teeth. Protibiae lacking an exterior lobe. Protibiae interior lobe spans the entire length of the protibiae and is not particularly wide, only about the same width as the protibial shaft itself. The lobe is smoothly triangular and is slightly wider towards the distal half. Mesotibiae and metatibiae lacking exterior and interior lobes.

###### Measurements of holotype

[**mm].** Length of body (including cerci and head, excluding antennae) 54.0, length/width of head 5.7/5.1, antennae 2.9, pronotum 4.0, mesonotum 2.7, length of tegmina 36.0, greatest width of abdomen 28.0, profemora 10.0, mesofemora 8.3, metafemora 9.9, protibiae 5.7, mesotibiae 6.4, metatibiae 8.2.

###### Etymology.

Noun. Named for the nymph Daphne of Greek mythology who was pursued tirelessly by the god Apollo and was eventually after pleading with her father for a way to escape the relentlessness of Apollo, was turned into a laurel tree. Derived from Greek, Δάφνη.

###### Distribution.

Currently only known from Biak Island, Papua Province, Indonesia.

#### Possible unconfirmed sexes of known *Nanophyllium* species/ notable specimens which cannot be identified to species

##### Nanophyllium

Taxon classificationAnimaliaPhasmidaPhylliidae

species (male)

87E7EBAB-8BFC-505D-ABED-45375D2C0707

Figure 22A

###### Collection data.

One male, observed and collected by Mike Wild (USA/Indonesia) in 2015. **Indonesia**: Papua Province, Puncak Jaya Regency, Mokndoma, around 2,180 meters elevation.

###### Discussion.

**Figure 22.**
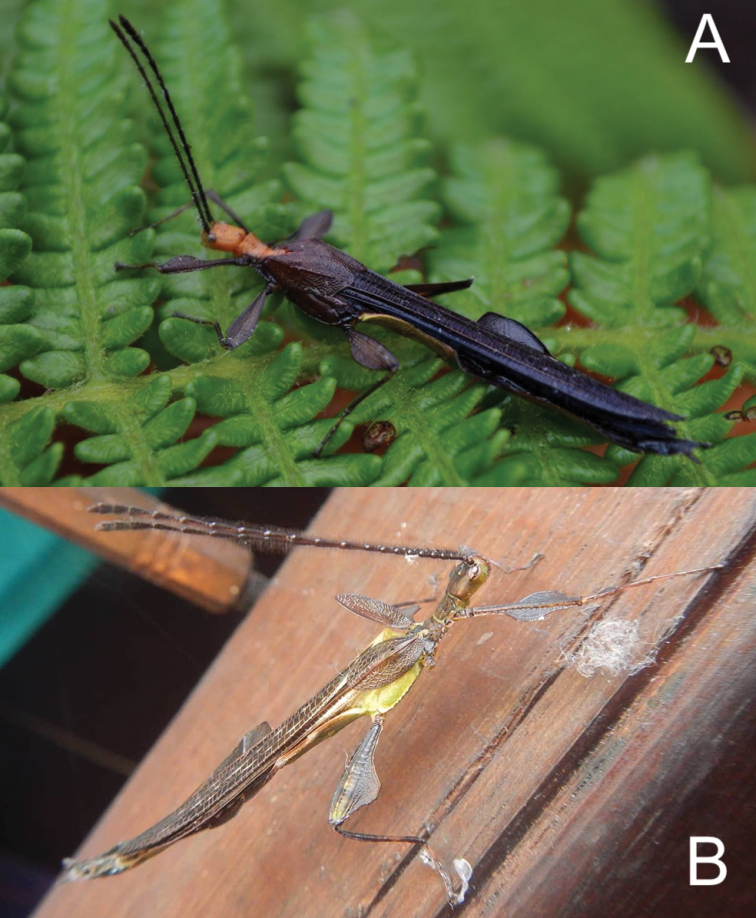
Live observations of unidentifiable male *Nanophyllium***A** individual observed by Mike Wild in Mokndoma, Indonesia **B** individual observed by Achmad Rian Dietra, May, 2017 on Aiduma Island, Indonesia.

This individual was observed and photographed by Mike Wild, who notes that despite living in the area for more than 14 years, and actively observing and collecting insects there the entire time, this is the only leaf insect he has ever seen. This species has highly reduced exterior profemoral lobes, which places it morphologically most similar to *N.australianum* (Fig. 11D) from Australia. This particular feature is not observed in other New Guinea known males to such a slender degree. This unknown species can be differentiated from *N.australianum* by the orange head, pronotum, and mesonotum (Fig. 22A) a unique feature in and of itself as all other known *Nanophyllium* males have the head and thorax the same color as the rest of the body. It is possible that this male may represent the unknown sex of one of the known female *Nanophyllium* or represent an undescribed species, but at this time it cannot be determined with so many species only known from a single sex.

Originally proposed by Rentz (1988) we agree that these darker and slightly metallic *Nanophyllium* males appear to not mimic foliage, but to instead be mimicking a wasp. Easily observed in this individual from Mokndoma and noted by Rentz (1988) the dark coloration is “shining black with a bluish overcast”. This coloration is common within Scoliidae and Pompilidae, both of which are large and intimidating wasps within the correct size range of a *Nanophyllium* male. Additionally, this particular specimen from Mokndoma has a bright orange head, pronotum, and mesonotum, and many species of these large wasps also have yellow, orange, or red segments of their bodies. We hope that examination of wasp species from this region and additional *Nanophyllium* males will help to identify possible species models.

##### Nanophyllium

Taxon classificationAnimaliaPhasmidaPhylliidae

species (male)

473C8348-1BC3-5007-B1C7-D76F588DB75E

Figure 22B

###### Observational collection data.

One male, observed by Achmad Rian Dietra (Indonesia) in May of 2017. **Indonesia**: West Papua Province, Kaimana Regency, Aiduma Island.

**Discussion.** This is only known from photographs of a live individual taken by Achmad Rian Dietra (Indonesia). Based on pro- and mesofemoral lobes being strongly angular and not smoothly arcing from end to end, this individual belongs to the *pygmaeum* species group. This species group only has males known for six species: *N.pygmaeum* Redtenbacher, 1906, *N.asekiense* (Größer, 2002), comb. nov., *N.adisi* Zompro & Größer, 2003, *N.rentzi* Brock & Größer, 2008, *N.hasenpuschi* Brock & Größer, 2008, and *N.australianum* Cumming, Le Tirant & Teemsma, 2018.

Based on the profemoral exterior lobe that is wider than the shaft width and not larger than the interior lobe, that rules out *N.australianum* (exterior lobe of profemora same width as shaft width; Fig. 11D) and *N.adisi* (exterior lobe of profemora larger than interior lobe; Fig. 11H). *Nanophylliumrentzi* (Fig. 18) and *N.asekiense* (Größer, 2002), comb. nov. (Fig. 17E, F), can also be ruled out as possible identifications, as their entire body coloration is green and the alae are completely transparent, in contrast this specimen from Aiduma Island has a brown body and dark tegmina and alae. The two remaining identification possibilities are *N.pygmaeum* and *N.hasenpuschi* which can easily be morphologically separated by the coloration of the alae, solid brown in *N.pygmaeum* or alae with a large transparent patch in *N.hasenpuschi*. Unfortunately, this individual has its wings closed so the interior color is impossible to see. A definitive identification is unfortunately not possible at this time. Geographically this individual is located near collection sites of both *N.hasenpuschi* and *N.pygmaeum* so no inference can be drawn from locality (Fig. 4). This is however a unique opportunity to share photos of a live individual and to add a new distribution checkpoint to the map of *Nanophyllium* collection/observation localities (Fig. 15). It is also possible that this individual represents an undescribed species on its own, or the male for an undescribed species based on one of the below females illustrated.

#### Presumed records for female *Nanophylliumaustralianum* Cumming, Le Tirant, & Teemsma, 2018

*Nanophylliumaustralianum* specimens are exceedingly rare (likely due to a lack of extensive collecting in the area they are found in), with only four collections/observations known to the authors to date and all known from in/near Iron Range National Park of Northern Queensland, Australia (Fig. 4).

**Figure 23.**
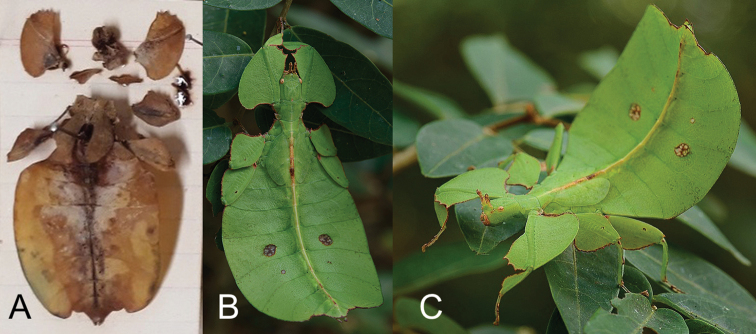
Likely *N.australianum* subadult females **A** preserved specimen which was collected by G. B. Monteith in June 1971 on the edge of Iron Range, Queensland (photograph by Susan Wright, collections manager, Queensland Museum) **B** dorsal, live individual photographed by Chien C. Lee (Malaysia) in July 2014; Lockhart, Queensland **C** same individual as in B but viewed from the lateral aspect.

The first known record is a subadult female discovered by G. B. Monteith in June 1971 while he was collecting along the edge of the rainforest of Iron Range. Monteith recognized this individual as a second species for Australia and likened the species to a specimen from Popondetta, Papua New Guinea (Fig. 23A; Monteith 1971). This species was again referenced in Key (1974) but this time as “*P.frondosum*” based on the subadult nymph collected by Monteith (Monteith 1978).

The second collection record we are aware of is an early instar nymph collected by G. B. Monteith in February 1976, near Gordon’s Mine Area, Iron Range (see Cumming et al. 2018: fig. 2 for an image of this nymph, note the shape of the femoral lobes which are angled with distinct teeth like which can be seen in the adults).

The third record was the holotype male which was collected as a nymph by D. C. F. Rentz near Mt. Tozer within the Iron Range in December 1986 and which matured to adult in January 1987 (see Rentz (1988) for notes and photographs of the nymph when it was caught, and see Cumming et al. (2018) for images of the resulting adult and species description).

The only other individual we are aware of is a subadult female which was photographed by Chien C. Lee in July 2014 in Lockhart, Queensland (Fig. 23B, C). This female matches the morphology of the first nymph which was collected as a subadult by Monteith in 1971, and with it observed near the same general collecting locality as the holotype male *N.australianum*, coupled with the similarity in femoral shapes to the male, we expect these females represent the female *Nanophylliumaustralianum*. The authors hope that future collection efforts in the Iron Range area will yield additional specimens so we can better review the intraspecific variation of this rarely collected species and allow the morphological description of the female adult morphology.

### Unidentified *Nanophyllium* species (females) (listed from the smallest to the largest specimens)

**Figure 24.**
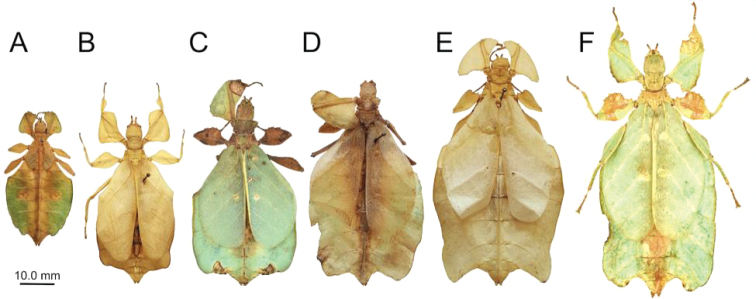
*Nanophyllium* females and their collection data which likely represent the unknown female *Nanophylliumrentzi*, *Nanophylliumhasenpuschi*, *Nanophylliumpygmaeum*, *Nanophylliumadisi*, or possibly undescribed species, scaled to relative size **A** Eastern Highlands District LJBrass, Coll., Sixth Archbold Exped. To Papua New Guinea, No. 7, Kotuni, south slopes Mt. Otto, 2200m. Aug.4–20.1959, AMNH**B** Papua New Guinea, Central Province, Along Hiritano HWY., E. of Vanama River Crossing: June 1989, Coll RC 16-224 **C** Papua New Guinea, Central, 20 km SE Port Moresby, I.1985, NHMUK 012497230 **D** Daru, Papua (New Guinea) Mouth of Fly R., VII-1941, Collector RG Wind, CAS**E** New Guinea, West Irian, Kobakma, North Slope of Central Range N. of Baliem Valley 3500’ October 1971, Robert Mitton Coll, CAS**F** Indonesia, West Papua, Mapia, May 1999, Coll SLT.

***Nanophyllium* sp. Female NYMPH (35 mm)**: Papua New Guinea: Eastern Highlands District LJBrass, Coll. Sixth Archbold Exped. To Papua New Guinea. No.7, Kotuni, south slopes Mt.Otto, 2200m. Aug. 4–20, 1959. (AMNH). (Fig. 24A).

This subadult could be *N.asekiense* or *N.frondosum* based on the geographic proximity to *N.asekiense* and *N.frondosum* known localities and how large it might be if it had reached adulthood. It is likely too large to have been one of the smaller species like *N.pygmaeum*.

***Nanophyllium* sp. Female (46.7 mm)**: Papua New Guinea: Central Province, Along Hiritano Highway, East of Vanapa River crossing. June 21^st^, 1989. Collected by L. M. Munsey, previously from the collection of Jerri Larsson (California), (Coll RC 16-224). (Fig. 24B).

**Habitat.** From the notes of L. M. Munsey the collector of the two specimens: “Daytime beating in a 1 to 3 acre area of cuttings with few small and large downed trees remaining”.

***Nanophyllium* sp. Female (54.0 mm)**: Papua New Guinea: Central Province, 20Km SE Port Moresby “bushes” 26.i.1985 J.W.Ismay. Ex Papua New Guinea DPI-CRIC Konedobu. C.I.E. COLL. A. 17440. NHMUK 012497230. (Fig. 24C).

***Nanophyllium* sp. Female (59.6 mm)**: Papua New Guinea: Daru, Papua (New Guinea) Mouth of Fly R. VII-1941. Collector RG Wind. Van Dyke Collection. (CAS). (Fig. 24D).

These three small adult females possibly represent the unknown female for *Nanophylliumpygmaeum* as they are the correct size and geographically are from southern Papua New Guinea, with the female from CAS from nearby the Fly River, which is the *N.pygmaeum* type locality.

***Nanophyllium* sp. Female (70.5 mm)**: Indonesia: New Guinea: West Irian, Kobakma, North Slope of Central Range N. of Baliem Valley 3500’ October 1971, Robert Mitton, Coll., Presented by H. Vannoy Davis, C.A.S. Accession. (CAS). (Fig. 24E).

Based on the size and morphology this female from Kobakma is similar to *N.frondosum* females which are known from other distant localities in Papua New Guinea. Unfortunately, the holotype *N.frondosum* has no other locality information other than “Neu-Guinea” (Redtenbacher, 1906) and therefore we cannot determine if *N.frondosum* is a wide-ranging species or if there is a complex of *N.frondosum*-like species. Hopefully future molecular comparison of freshly collected material can reveal the extent of *N.frondosum*.

An additional possibility is that this female could be the opposite sex of *Nanophylliumadisi*, which is known from nearby this collection location from “Hoofdbivak, 250 m Datum IX” from the Stirling Expedition of 1926 (present day Indonesia: Papua Province, Nduga Regency). This possibility is in our opinion unlikely as these localities are separated by the expansive Maoke Mountains which are most likely a geographic barrier between these populations.

***Nanophyllium* sp. Female (75.0 mm)**: Indonesia, West Papua, Mapia, V.1999 (Coll SLT) (Fig. 24F).

This female is from very near the type locality of *N.hasenpuschi* and could possibly represent the unknown female. Hopefully the holotype *N.hasenpuschi* can be sequenced one day to be compared and possibly matched with this female. Morphologically this female is similar to *N.asekiense* as it has slight exterior tibial lobes and similar prominent serration of the femoral lobes.

#### Biogeography

Figures 4, 15

Phylliidae arose according to Bradler et al. (2015) in the Eocene approximately 55–65 mya ago. However, other phylogenetic approaches (Goldberg et al. 2015; Büscher et al. 2018a, b; Robertson et al. 2018) discuss monophyletic Phylliidae in various different relationships to other groups, which would suggest a later origin of the lineage. The early radiation of the extant *Phyllium* and *Chitoniscus* species is dated ~30–35 mya ago (Bradler et al. 2015; Robertson et al. 2018). The report of the fossil *Eophylliummesselense* Wedmann, Bradler & Rust, 2007 gives further indication that Phylliidae arose more than 47 mya ago and estimates the splitting of *Nanophyllium* from the remaining Phylliidae ~ 20 mya ago (Wedmann et al. 2007). Assuming an approximate splitting of *Nanophyllium* 20–35 mya from the remaining Phylliidae some conclusions on the biogeographical history of *Nanophyllium* can be drawn. Ancestors of *Nanophyllium* probably settled New Guinea close to that timeframe, after New Guinea emerged in the Eocene (~ 40 mya) and was separated from Australia during the Oligocene (~25 mya) after the New Guinea Passive Margin collided with the leading edge of the Eastern Philippines-Halmahera-New Guinea Arc System (Hall 2001). As extant phylliids inhabit most parts of the Oriental and Australian region, but *Nanophyllium*, so far known, is restricted to New Guinea, including the surrounding islands, and Australia (Fig. 4), this lineage probably evolved on New Guinea and later migrated to northern Australia. *Nanophyllium* likely diversified on New Guinea due to geographic isolation by the Central Cordillera (Chisholm 1911) and the Foja Mountain Ranges in the north (Richards et al. 2009; Oliver et al. 2011). During the Pleistocene (2.6 mya–11.7 ka) Australia and New Guinea were interconnected many times, due to climatic oscillation resulting in sea level fluctuations (Pillans et al. 1998), but the remaining parts of Indonesia remained separated from New Guinea (Ludt and Rocha 2015). The southernmost population of *Nanophyllium* possibly distributed to Australia and became separated, resulting in the speciation of *N.pygmaeum* and *N.australianum* after the Last Glacial Maximum ~ 17–19 ka ago (Ludt and Rocha 2015). The distributional records of the known *Nanophyllium* specimens suggest a well-established isolation of the distributions of *N.miyashitai* sp. nov. from other *Nanophyllium* by the Central Cordillera. As this formation arose quite early (probably during the Oligocene (Hall 2001), this population potentially has been isolated for a comparatively long time, that likely led to allopatric speciation. Likewise, most of the other *Nanophyllium* species are probably a result of allopatric isolation caused by this mountain range. *Nanophylliumkeyicum* and *N.daphne* sp. nov. in contrast, are probably a result of the isolation on the islands they are found, which became separated from mainland New Guinea.

### Key to known *Nanophyllium* males^*^

**Table:** 

1	Profemoral interior lobes which are rounded without a sharp angle; mesofemoral interior lobes which are a large rounded triangle, reaching from end to end without prominent spination; (alae) the media anterior and the media posterior do no fuse, instead they both run to the wing margin, and the cubitus after splitting from the first anterior anal fuses with the media posterior near the wing margin and then they run fused to the margin as one; (*stellae* species group)	1
–	Profemoral interior lobe angular; mesofemoral interior lobes which do not reach from end to end of the shaft and have distinct serrate teeth; (alae) the media posterior fuses back to the media anterior before reaching the wing margin, and then the fused media runs on its own to the wing margin without fusing with the radial sector; (*pygmaeum* species group)	4
2	Mesopleurae with a single anterior tubercle, remainder lacking tubercles; tegmina longer (almost the length of the metathorax)	3
–	Mesopleurae with a prominent anterior tubercle followed by four additional small tubercles; tegmina shorter (only about half the length of the metathorax)	*N.miyashitai* sp. nov.
3	Exterior profemoral lobe smoothly rounded with an obtuse angle; abdominal segments with smooth edges creating a clean, spade-shaped abdomen	*N.stellae*
–	Exterior profemoral lobe slightly recurved creating an overall acute angle; abdominal segment V with two large clear spots; segments V–VII each with margins that extend and then contract creating a scalloped edge	*N.larssoni*
4	Alae completely transparent, not fully brown in color or with a brown band along the margin; tegmina transparent	5
–	Alae color either completely brown or with a transparent center and brown margin; tegmina completely brown	6
5	Profemoral interior lobe notably larger than the exterior lobe	*N.asekiense*
–	Profemoral interior lobe equal width to the exterior lobe	*N.rentzi*
6	Exterior profemoral lobe distinct, wider than the width of the profemoral shaft	7
–	Exterior lobe of profemora greatly reduced, not wider than the width of the profemoral shaft	*N.australianum*
7	Exterior profemoral lobe notably tapered on the distal and proximal ends; the interior profemoral lobe can be of the same size as the exterior lobe or larger than the exterior lobe	8
–	Exterior profemoral lobe only notably tapered on the proximal end, with the distal nearly reaching the end of the profemoral shaft; profemoral interior lobe always smaller than the exterior lobe	*N.adisi*
8	Alae almost completely brown, or completely brown in color	*N.pygmaeum*
–	Only the alae margin and sclerotized section brown, interior half of the alae transparent	*N.hasenpuschi*

### Key to known Nanophyllium females

**Table:** 

1	Small species (~ 56.0 mm or less); (tegmina) the radial bend occurs before the splitting of the first radial and the radial sector, therefore the radial sector is straight; the radius and medial crossvein is present on the radial bend at or before the splitting of the first radial	**2**
–	Larger species (> 56.0 mm); (tegmina) the bend in the radial vein happens on the radial sector after the splitting of the first radial from the radius; the radius and medial crossvein occurs after the splitting of the first radial, instead originating on the radial sector	**3**
2	Profemoral exterior lobe broad, with a slight recurve, giving the exterior angle an acute or right angle; mesofemoral interior lobe with the widest portion on the proximal half	***N.chitoniscoides* comb. nov.**
–	Profemoral exterior lobe narrow, smoothly arcing from end to end with the exterior angle distinctly obtuse; mesofemoral interior lobe with the widest portion on the distal half	***N.daphne* sp. nov.**
3	(Tegmina) there is a wide gap between the media and cubitus veins which persists throughout their entire length, this gap is several times wider than a single vein width; profemoral exterior lobe proximal margin is straight, not recurved	***N.keyicum* comb. nov.**
–	(Tegmina) the media and cubitus veins run side by side throughout the entire length either touching or no farther than a single vein width apart; profemoral exterior lobe proximal margin is recurved, not straight	**4**
4	Prescutum width more than two times the length; mesofemoral exterior lobe broad, notably wider than the mesofemoral shaft	***N.suzukii* comb. nov.**
–	Prescutum width less than two times the length; mesofemoral exterior lobe as wide as or thinner than the mesofemoral shaft	**5**
5	No protibial exterior lobes and no mesotibial exterior lobes, exteriors simple	***N.frondosum* comb. nov.**
–	Distinct protibial exterior lobes and mesotibial exterior lobes, present as small spurs	***N.asekiense* comb. nov.**

## Conclusions

Review of a wide number of institution and private collections as well as the successful rearing by the Montreal Insectarium has revealed the identity of a previously unconfirmed female *Nanophyllium*. This has allowed us to synonymize the *frondosum* species group (only known from females) with the *Nanophyllium* (only known from males) into a single genus.

Unfortunately, due to the striking sexual dimorphism in *Nanophyllium*, this leaves many females and males with unknown opposite sexes and the possibility that some of the presently described species of either group might simply be the opposite sex of an already known species. Hopefully future collections of fresh material from throughout the region will either allow successful rearing of species to elucidate the unknown sex or allow pairing of sexes on a molecular basis. Additionally, we eagerly await extensive molecular analysis for the Phylliidae as a whole to elucidate the higher taxonomy within the family and the placement of the *Nanophyllium*.

## Supplementary Material

XML Treatment for Nanophyllium asekiense

XML Treatment for Nanophyllium miyashitai

XML Treatment for Nanophyllium daphne

XML Treatment for Nanophyllium

XML Treatment for Nanophyllium

## Footnotes

Adapted from Cumming et al. 2018.
